# Impact of the COVID-19 pandemic on health emergency and disaster risk management systems: a scoping review of mental health support provided to health care workers

**DOI:** 10.1093/joccuh/uiaf020

**Published:** 2025-03-31

**Authors:** Jargalmaa Amarsanaa, Oyundari Batsaikhan, Badamtsetseg Jargalsaikhan, Tatsuhiko Kubo, Nader Ghotbi, Ryoma Kayano, Odgerel Chimed-Ochir

**Affiliations:** Department of Public Health and Health Policy, Graduate School of Biomedical and Health Sciences, Hiroshima University, Kasumi 1-2-3, Minamiku, Hiroshima 734-8553, Japan; Department of Public Health and Health Policy, Graduate School of Biomedical and Health Sciences, Hiroshima University, Kasumi 1-2-3, Minamiku, Hiroshima 734-8553, Japan; Department of Public Health and Health Policy, Graduate School of Biomedical and Health Sciences, Hiroshima University, Kasumi 1-2-3, Minamiku, Hiroshima 734-8553, Japan; Department of Public Health and Health Policy, Graduate School of Biomedical and Health Sciences, Hiroshima University, Kasumi 1-2-3, Minamiku, Hiroshima 734-8553, Japan; College of Asia Pacific Studies, Ritsumeikan Asia Pacific University, 1-1 Jumonjibaru, Beppu, Oita 874-8577, Japan; WHO Centre for Health Development, I.H.D. Centre Building, 9th Floor, 1-5-1 Wakinohama-Kaigandori, Chuo-ku, Kobe 651-0073, Japan; Department of Public Health and Health Policy, Graduate School of Biomedical and Health Sciences, Hiroshima University, Kasumi 1-2-3, Minamiku, Hiroshima 734-8553, Japan

**Keywords:** COVID-19, psychosocial support systems, mental health, health personnel, disaster planning

## Abstract

**Objectives**: This systematic scoping review examined the strategies used by different countries and institutions to support the mental health of health care workers (HCWs) during the COVID-19 pandemic, to identify effective practices and the lessons learned in dealing with the associated challenges.

**Methods**: Of 1330 retrieved articles from PubMed, Scopus, and the Web of Science, 34 articles were ultimately included in the final analysis.

**Results**: The analysis revealed that mental health consultation services, especially telephone support lines, online interventions, and apps, played a critical role in addressing the psychological burden experienced by HCWs. Group activities and peer support strategies offered personalized support, and educational programs offered crucial information regarding stress management. Improvements in the work environment, such as the addition of dedicated rest areas, enhanced the well-being of HCWs. However, many interventions suffered from low participation and a lack of tailored content, despite their apparent effectiveness.

**Conclusions**: Many interventions have focused on psychological support and resilience-building for HCWs, but they often overlook systemic issues. Comprehensive mental health support must address these systemic factors, such as adequate staffing, training, and resource allocation. Future strategies should emphasize leadership commitment to tackling root causes and actively involve HCWs in program design to ensure relevance and effectiveness. Educational resources and wellness interventions, although reported as effective, need to be tailored and adapted to specific emergencies. Additionally, research gaps, especially in low-resource settings, highlight the need for further studies to enhance preparedness for future crises.

## Teaser

Key points

- **What is already known on this topic:** It is well established that the COVID-19 pandemic imposed severe challenges on health care systems worldwide, with health care workers being particularly vulnerable to psychological stress due to high patient loads, direct exposure to the virus, and the emotional toll of caring for patients. Academic studies have documented the numerous mental health programs that were implemented globally to support health care workers. However, there was a lack of synthesized knowledge regarding the effectiveness of these strategies, and best practices for managing the mental health of health care workers in future health crises.
- **What this study adds:** This study fills the gap in understanding the effectiveness of various mental health interventions used during the COVID-19 pandemic, highlighting both successful strategies and areas needing improvement. It identifies key best practices, such as ensuring basic needs, maintaining confidentiality, offering psychoeducation, and involving health care workers in program design, which are critical for developing effective mental health support systems.
- **How this study might affect research, practice, or policy:** In terms of policy, it guides policymakers to advocate for comprehensive, sustainable mental health strategies and dedicated support systems within health care settings to improve resilience for future crises. It also provides best practices for developing effective mental health support programs, including addressing basic needs, ensuring confidentiality, and involving health care workers in design. This research suggests the need for health care organizations to shift from individual-focused interventions to systemic strategies addressing root causes of health care workers’ stress and burnout.

## 1. Introduction

The COVID-19 pandemic imposed unprecedented challenges on health care systems, economies, and societies throughout the world, and could be considered a disaster situation, particularly during the early stages.^1^ Health care systems worldwide faced immense pressure to mount emergency responses to the pandemic.^2^ Throughout the pandemic, health care workers (HCWs) were at the forefront of the response, and occupational health management systems played a pivotal role in safeguarding frontline HCWs, other health care professionals, and other essential personnel.^3^ The experiences of these individuals during the COVID-19 pandemic highlighted the critical importance of providing mental health support for HCWs.^4^ The unprecedented challenges faced by doctors and nurses, including high patient loads, exposure to the virus, and the emotional toll of witnessing patients suffering and dying, necessitated a comprehensive response to address their psychological well-being.^5^

Although numerous academic studies have described the programs used to manage the mental health of HCWs,^6^^,^^7^ little is known about the lessons to be learned from these programs and the best practices to be used for future crises. As we approach nearly  5 years since the beginning of the COVID-19 pandemic, data about the responses of different health care systems are now available, underscoring the need to identify the lessons learned and to synthesize the best practices regarding the mental health interventions to be used to assist health care professionals.

A comprehensive assessment of the occupational mental health measures that were implemented during the pandemic can shed light on the effectiveness of different strategies, and help to improve preparedness in future health emergencies. Thus, the primary objective of this study was to analyze the many different interventions during the COVID-19 pandemic that targeted the mental health of HCWs, and to identify the lessons learned and best practices.

The World Health Organization (WHO) developed the Health Emergency and Disaster Risk Management (Health EDRM) Framework,^8^ and this Framework offers guidance to countries and partners to help them mitigate the risks and impacts of all types of emergencies and disasters, including epidemics and pandemics. This framework has 10 components and functions, and considers human resources as critical for enhancing a country’s resilience when facing a disaster. Within the Health EDRM framework, the key considerations in human resource management include planning for changes in staffing; developing surge capacity for emergency responses; and implementing additional education and training programs to improve the competence, occupational health, and safety of health care workers. Our previous study examined the responses of different countries when there were sudden needs for changes in staffing.^9^ The present scoping review analyzed the strategies employed by various institutions across different countries during the COVID-19 pandemic that were designed to address the mental health issues faced by HCWs.

## 2. Methods

The design of this scoping review framework was based on the Joanna Briggs Institute (JBI) framework of evidence synthesis,^10^ which consists of 5 stages: (1) identifying the research question, (2) identifying relevant studies, (3) selecting studies, (4) presenting the data, and (5) collating the results.

### 2.1. Identifying the research question

The JBI Framework says that the review question should include information on the “participants,” the main focus or “concept,” and the “context” of the review (PCC, Participant, Concept, Context). In the current review, the “participants” are HCWs, the “concept” is the response of different countries and institutions to the mental health challenges faced by HCWs, and the “context” is the COVID-19 pandemic. Based on this PCC framework, the following research questions were developed:

- What were the responses of different countries and institutions to the mental health issues faced by HCWs during the COVID-19 pandemic?
- Were these responses perceived as effective?
- What were the perceived advantages and best practices of these responses?
- What were the perceived disadvantages and lessons learned from these responses?

### 2.2. Identifying relevant studies

We searched for relevant scientific papers in 3 electronic databases: PubMed, Web of Science, and Scopus. The basic search consisted of the key terms “Healthcare workforce” AND “COVID-19” AND “Mental health”. A complete description of the search strategy is given in Table S1. Table 1 shows the criteria used for inclusion and exclusion of different studies.

**Table 1:** Inclusion criteria of the reviewed articles.

	**Inclusion criteria**	**Exclusion criteria**
**Population**	Health care workers (HCWs: including physicians, nurses, allied health professionals, and support staff) working in any health care setting (including hospitals, primary care clinics, and community health centers) during the COVID-19 pandemic	Non-HCWs
**Concept**	Any type of responses to support the mental health of HCWs	Only highlighting the mental well-being or health problems of HCWs but not addressing the response to these issues
**Context**	COVID-19 pandemic, including any phase of the pandemic (eg, initial outbreak, subsequent waves, vaccination campaigns)	Non–COVID-19 infection including SARS, MERS
**Publication type**	All types of publications about responses to mental health issues of HCWs during the COVID-19 pandemic and published in peer-reviewed journals	Articles in preprint server, other publication venues, including books, book chapters, and gray literature
**Publication date**	1 January 2020 to 8 September 2023	NA
**Study design**	Any study design, including empirical and nonempirical works that answer the research questions	Clinical randomized trials
**Language**	English	Other than English

Abbreviations: HCW, health care worker; MERS, Middle East respiratory syndrome; NA, not applicable; SARS, severe acute respiratory syndrome.

### 2.3. Study selection

After removing duplicates from the initial search results, an initial screening of the titles and abstracts was conducted independently by two researchers (O.C.-O. and J.A.). When these researchers did not agree on the eligibility of a study, the publication was subjected to full-text review prior to making a decision. After elimination of ineligible articles, the full text of each included article was screened. All relevant references that were cited in these included articles were also reviewed to identify additional articles. This search was reported using the Preferred Reporting Items for Systematic reviews and Meta-Analysis (PRISMA) flow chart.^11^

**Figure 1:**
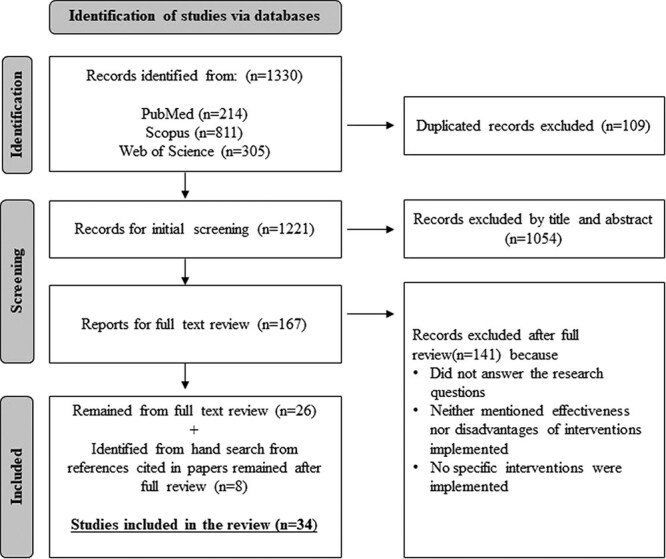
PRISMA (Preferred Reporting Items for Systematic reviews and Meta-Analysis ) flowchart.

**Figure 2:**
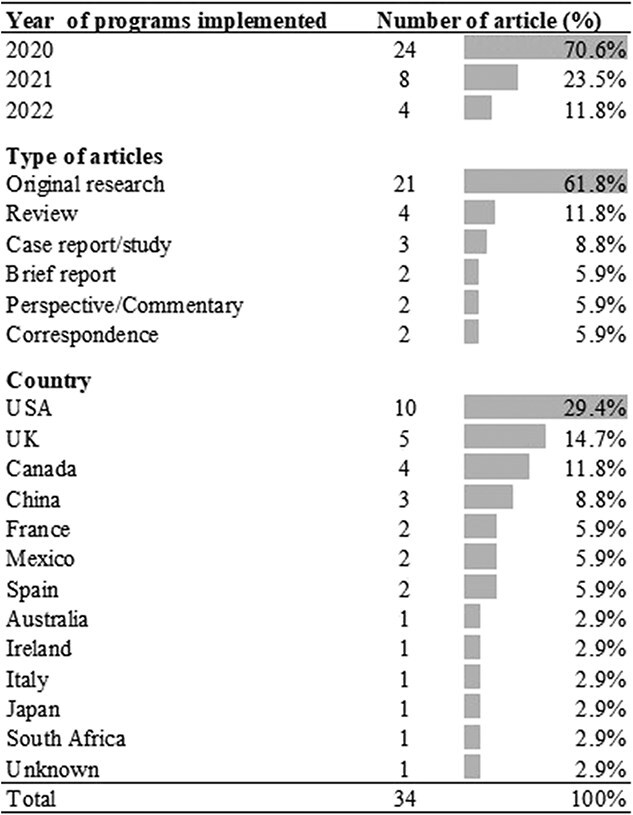
General bibliographic information of reviewed articles.

### 2.4. Data presentation

Two authors (O.C.-O. and A.J.) extracted the following information from all articles: authors, title, publication date, period covered by the study, study design, type of responses to mental health issues, advantages/best practices and disadvantages/lessons learned from these responses, and country of the study. The 2 independent researchers summarized the data from the included articles in an Excel spreadsheet using these categories. When these 2 researchers could not reach agreement, a third researcher (T.K.) adjudicated.

### 2.5. Collating the results

A summary of basic bibliometric information was presented for all included studies. Then, a qualitative narrative synthesis was performed to analyze the characteristics of the different studies.

## 3. Results

We initially retrieved 1330 articles from 3 databases (PubMed, Scopus, and Web of Science). After removing duplicate articles and screening of titles and abstracts, we identified 167 articles for full review. After the full-text review, there were 27 articles remaining. We then searched for relevant articles from the references cited in these 27 papers. Finally, we identified 34 articles that were eligible for analysis (Figure 1).

Among the 34 included articles, 24 (70.6%) were conducted in 2020, and 21 (61.8%) were original research articles. These articles were from 17 different countries, and 10 (29.4%) were from the United States (Figure 2). Table 2 shows the detailed bibliographic information for all 34 articles.

We classified these studies according to the type of intervention: (1) mental health consultation; (2) group activities and peer support; (3) educational program; (4) improvement of the work environment and basic needs; and (5) others. The summary of findings is presented in Table 3, and detailed information for each article is provided in Table S2.

### 3.1. Mental health consultation

The mental health consultation services provided to HCWs are pivotal for addressing the psychological burdens the HCWs experience during stressful periods. During the COVID-19 pandemic, these services were provided in various forms, including telephone support lines,^12-17^ online multicomponent psychological interventions,^18-20^ video conferencing,^12^^,^^21^^,^^22^ video debriefing sessions,^22^ and apps that offered individualized resources.^23^^,^^24^ The overall positive evaluations of these interventions highlight their critical role in supporting the mental well-being of HCWs. For example, several apps were developed soon after the pandemic started, and the psychological resources available through these apps, such as group chat and specific QR codes to provide information on available resources, provided timely assistance to a large number of HCWs. These apps, including Heroes Health, Be + against COVID,^23^ Wellness Hub, Clinicovery, and PsyCovidApp,^15^ provided services such as self-assessment, recommendations, and different types of support resources, including relaxation videos, resilience-building exercises, and brief daily questionnaires to evaluate stress. Moreover, some apps provided support for HCWs through hotline telephone numbers and support groups via videoconferencing.^22^ One study described a Healthcare Worker Mental Health COVID-19 Hotline that provided crisis counseling by psychiatrists, clinical social workers, psychiatric residents, and volunteer mental health professionals.^14^

#### 3.1.1. Advantages and best practices

The accessibility and convenience of these services were reported as their major advantages.^12^^,^^15^^,^^17^^,^^21^ Telephone support lines and online platforms allowed HCWs to access help whenever needed, and they could even leave voice messages, in an effort to provide continuous support.^16^^,^^21^ These services were crucial for promptly identifying and addressing mild symptoms of anxiety, and referring individuals to the most appropriate services.^14^ The user-friendly nature of these platforms, regardless of internet availability, enhanced their reach and usability.^18^ Collection of opinions and feedback from HCWs was perceived as one of the most effective interventions, and organizations that developed mental health initiatives based on the needs and preferences of HCWs experienced better engagement and outcomes.^12^^,^^13^ In addition to these benefits, the ability of HCWs to connect with family and friends via social media platforms was very helpful.^25^

Video group therapy and video conference platforms increased the availability and access to mental health support.^19^^,^^20^ These interventions were also reported as effective when conducted in safe environments with trusted colleagues, because this fostered a sense of community and support among participants.^21^^,^^22^ Additionally, online programs that offered monitoring and interventions enabled managers to assess the mental health of HCWs so they could offer weekly psychological support to ensure the well-being of their employees.^12^^,^^19^

#### 3.1.2. Disadvantages and lessons learned

Despite the advantages of these consultation services, there were several challenges and lessons to be learned. The highly intense work schedules during the pandemic often limited the ability of HCWs to access these services.^12^^,^^19^ Video conference and debriefing programs were reported to be only effective for HCWs who were willing and able to share their issues and vulnerabilities with peers and other colleagues.^22^ Additionally, these interventions were unsuitable for the diagnosis or treatment of HCWs with serious mental health problems,^15^^,^^18^ so were only beneficial to those with less serious psychological problems.^21^ The lack of recorded caller feedback and satisfaction metrics for hotlines and other services prevented a comprehensive evaluation of their effectiveness.^15^ Some authors also pointed out the need to implement psychological interventions that had a more scientific basis, so that their effectiveness could be more readily determined.^20^^,^^24^ The need for additional human resources to sustain these services in the long run was another concern.^14^

**Table 2:** Bibliographic information on articles reviewed.

**#**	**Author information**	**Article title**	**Journal published**	**Article type**	**Study covering period**	**Publication year**	**Country**
1^35^	d’Ussel M, Adam F, Fels A, Chatellier G, Philippart F	Characteristics of hospital workers using a wellbeing center implemented during the COVID-19 pandemic to prevent the emotional impacts of the crisis	*Frontiers in Public Health*	Community case study	July and October 2020	2022	France
2^30^	Rosen B, Preisman M, Read H, Chaukos D, Greenberg RA, Jeffs L, Maunder R, Wiesenfeld L	Resilience coaching for healthcare workers: Experiences of receiving collegial support during the COVID-19 pandemic	*General Hospital Psychiatry*	Original article	April 2020	2022	Canada
3^12^	Martindale SL, Shura RD, Cooper MA, Womack SF, Hurley RA, Vair CL, Rowland JA	Operational stress control service: an organizational program to support health care worker well-being	*Journal of Occupational and Environmental Medicine*	Original article	April 2020 to February 2021	2022	United States
4^37^	Petrella AR, Hughes L, Fern LA	Healthcare staff well-being and use of support services during COVID-19: a UK perspective	*General Psychiatry*	Original article	April 2020	2021	United Kingdom
5^25^	Norful AA, Rosenfeld A, Schroeder K, Travers JL, Aliyu S	Primary drivers and psychological manifestations of stress in frontline healthcare workforce during the initial COVID-19 outbreak in the United States	*General Hospital Psychiatry*	Qualitative study	April 2020	2021	United States
6^22^	Monette DL, Macias-Konstantopoulos WL, Brown DFM, Raja AS, Takayesu JK	A video-based debriefing program to support emergency medicine clinician well-being during the COVID-19 pandemic	*Western Journal of Emergency Medicine*	Brief research report	March to April 2020	2020	United States
7^14^	Feinstein RE, Kotara S, Jones B, Shanor D, Nemeroff CB	A health care workers mental health crisis line in the age of COVID-19	*Depression and Anxiety*	Review	June 2020	2020	United States
8^38^	Zhu Z, Xu S, Wang H, Liu Z, Wu J, Li G, Miao J, Zhang C, Yang Y, Sun W, Zhu S, Fan Y, Chen Y, Hu J, Liu J, Wang W	COVID-19 in Wuhan: sociodemographic characteristics and hospital support measures associated with the immediate psychological impact on healthcare workers	*EClinicalMedicine*	Original article	February 2020	2020	China
9^15^	Geoffroy PA, Le Goanvic V, Sabbagh O, Richoux C, Weinstein A, Dufayet G, Lejoyeux M	Psychological support system for hospital workers during the Covid-19 outbreak: rapid design and implementation of the Covid-Psy hotline	*Frontiers in Psychiatry*	Original article	March to April 2020	2020	France
10^32^	Blake H, Bermingham F, Johnson G, Tabner A	Mitigating the psychological impact of COVID-19 on healthcare workers: a digital learning package	*International Journal of Environmental Research and Public Health*	Case study	April 2020	2020	United Kingdom
11^33^	Robles R, Palacios M, Rangel N, Real T, Becerra B, Fresán A, Vega H, Rodríguez E, Durand S, Madrigal E	A qualitative assessment of psycho-educational videos for frontline COVID-19 healthcare workers in Mexico	*Salud Mental*	Original article	May 2020	2020	Mexico

(Continued)

**Table 2:** Continued

**#**	**Author information**	**Article title**	**Journal published**	**Article type**	**Study covering period**	**Publication year**	**Country**
12^23^	Mira JJ, Vicente MA, Lopez-Pineda A, Carrillo I, Guilabert M, Fernández C, Pérez-Jover V, Martin Delgado J, Pérez-Pérez P, Cobos Vargas A, Astier-Peña MP, Martínez-García OB, Marco-Gómez B, Abad Bouzán C	Preventing and addressing the stress reactions of health care workers caring for patients with COVID-19: development of a digital platform (Be + against COVID)	*JMIR mHealth and uHealth*	Original article	March 2020	2020	Spain
13^40^	Mira JJ, Cobos-Vargas Á, Astier-Peña MP, Pérez-Pérez P, Carrillo I, Guilabert M, Pérez-Jover V, Fernández-Peris C, Vicente-Ripoll MA, Silvestre-Busto C, Lorenzo-Martínez S, Martin-Delgado J, Aibar C, Aranaz J	Addressing acute stress among professionals caring for COVID-19 patients: lessons learned during the first outbreak in Spain (March–April 2020)	*International Journal of Environmental Research and Public Health*	Original article	March 2020	2021	Spain
14^34^	Collins GB, Ahluwalia N, Arrol L, Forrest N, McGlennan A, O’Brien B, Proudfoot A, Trainer M, Schilling R, Sullivan E, Westwood M, Wragg A, Knight C	Lessons in cognitive unloading, skills mixing, flattened hierarchy and organisational agility from the Nightingale Hospital London during the first wave of the SARS-CoV-2 pandemic	*BMJ Open Quality*	Narrative review	2020	2021	United Kingdom
15^13^	Chen Q, Liang M, Li Y, Guo J, Fei D, Wang L, He L, Sheng C, Cai Y, Li X, Wang J, Zhang Z	Mental health care for medical staff in China during the COVID-19 outbreak	*Lancet Psychiatry*	Correspondence	February 2020	2020	China
16^17^	Lijun Kang, Yi Li, Shaohua Hu, Min Chen, Can Yang, Bing Xiang Yang, Ying Wang, Jianbo Hu, Jianbo Lai, Xiancang Ma, Jun Chen, Lili Guan, Gaohua Wang, Hong Ma, Zhongchun Liu	The mental health of medical workers in Wuhan, China dealing with the 2019 novel coronavirus	*Lancet Psychiatry*	Correspondence	January 2020	2020	China
17^39^	Ripp J, Peccoralo L, Charney D	Attending to the emotional well-being of the health care workforce in a New York City health system during the COVID-19 pandemic	*Journal of the Association of American Medical Colleges*	Commentary	March 2020	2020	United States
18^16^	Maldonato NM, Bottone M, Chiodi A, Continisio GI, De Falco R, Duval M, Muzii B, Siani G, Valerio P, Vitelli R	A mental health first aid service in an Italian university public hospital during the coronavirus disease 2019 outbreak	*Sustainability*	Case report	March to May 2020	2020	Italy
19^36^	Blake H, Yildirim M, Wood B, Knowles S, Mancini H, Coyne E, Cooper J	COVID-Well: evaluation of the implementation of supported wellbeing centres for hospital employees during the COVID-19 pandemic	*International Journal of Environmental Research and Public Health*	Original article	April to July 2020	2020	United Kingdom

(Continued)

**Table 2:** Continued

**#**	**Author information**	**Article title**	**Journal published**	**Article type**	**Study covering period**	**Publication year**	**Country**
20^21^	Viswanathan R, Myers MF, Fanous AH	Support groups and individual mental health care via video conferencing for frontline clinicians during the COVID-19 pandemic	*Psychosomatics*	Perspective	March 2020	2020	United States
21^26^	McLean CP, Betsworth D, Bihday C, et al	Helping the helpers: adaptation and evaluation of stress first aid for healthcare workers in the Veterans Health Administration during the COVID-19 pandemic	*Workplace Health Safety*	Original research	April to July 2021	2023	United States
22^29^	Simms L, Ottman KE, Griffith JL, et al	Psychosocial peer support to address mental health and burnout of health care workers affected by COVID-19: a qualitative evaluation.	*International Journal of Environmental Research and Public Health*	Original research	Not available	2023	United States
23^27^	Maunder RG, Kiss A, Heeney N, et al	Randomized trial of personalized psychological feedback from a longitudinal online survey and simultaneous evaluation of randomized stepped wedge availability of in-person peer support for hospital staff during the COVID-19 pandemic	*General Hospital Psychiatry*	Original research	Fall 2020 to Spring 2022	2023	Canada
24^41^	Kelsey EA	Joy in the workplace: the Mayo Clinic experience	*American Journal of Lifestyle Medicine*	Analytic review	Not available	2021	United States
25^24^	Hoedl M, Osmancevic S, Thonhofer N, Reiter L, Schoberer D	Psychosocial interventions for healthcare workers during the COVID-19 pandemic: rapid review and meta-analysis	*Wiener Medizinische Wochenschrift*	Rapid review and meta-analysis	Jan 2020 to June 2021	2023	
26^42^	Gerbarg P, Dickson F, Conte VA, Brown RP	Breath-centered virtual mind–body medicine reduces COVID-related stress in women healthcare workers of the Regional Integrated Support for Education in Northern Ireland: a single group study	*Frontiers in Psychiatry*	Original research	Dec 2020 to April 2021	2023	Ireland
27^48^	Havaei F, MacPhee M, Ma A, Wong VW, Li C, Cheung I, Scigliano L, Taylor A	Implementation of the Synergy tool: a potential intervention to relieve health care worker burnout	*International Journal of Environmental Research and Public Health*	Original research	2022	2022	Canada
28^18^	Dominguez-Rodriguez A, Martínez-Arriaga RJ, Herdoiza-Arroyo PE, Bautista-Valerio E, de la Rosa-Gómez A, Castellanos Vargas RO, Lacomba-Trejo L, Mateu-Mollá J, Lupercio Ramírez MJ, Figueroa González JA, Ramírez Martínez FR	E-Health psychological intervention for COVID-19 healthcare workers: protocol for its implementation and evaluation	*International Journal of Environmental Research and Public Health*	Original research	16 July 2021 to ongoing	2022	Mexico

(Continued)

**Table 2:** Continued

**#**	**Author information**	**Article title**	**Journal published**	**Article type**	**Study covering period**	**Publication year**	**Country**
29^19^	Chandler AB, Wank AA, Vanuk JR, O’Connor MF, Dreifuss BA, Dreifuss HM, Ellingson KD, Khan SM, Friedman SE, Athey A	Implementing psychological first aid for healthcare workers during the COVID-19 pandemic: a feasibility study of the ICARE model	*Journal of Clinical Psychology in Medical Settings*	Original research	May to July 2020	2023	United States
30^28^	Korman MB, Steinberg R, Gagliardi L, Stewart B, Acero CL, Davies J, Maunder R, Walker T, DasGupta T, DiProspero L, Sinyor M, Ellis J	Implementing the STEADY wellness program to support healthcare workers throughout the COVID-19 pandemic	*Healthcare (Basel)*	Brief report	March to October 2020	2022	Canada
31^31^	Olcoń K, Allan J, Fox M, Everingham R, Pai P, Keevers L, Mackay M, Degeling C, Cutmore SA, Finlay S, Falzon K	Narrative inquiry into the practices of healthcare workers’ wellness program: the SEED experience in New South Wales, Australia.	*International Journal of Environmental Research and Public Health*	Original research	May 2021 to March 2022	2022	Australia
32^43^	Miyoshi T, Ida H, Nishimura Y, Ako S, Otsuka F	Effects of yoga and mindfulness programs on self-compassion in medical professionals during the COVID-19 pandemic: an intervention study	*International Journal of Environmental Research and Public Health*	Original research	January to March and May to July 2021	2022	Japan
33^44^	Osman I, Singaram V	Using PhotoVoice to understand mindfulness in health care practitioners.	*Health SA*	Original research	June to August 2020	2022	South Africa
34^20^	Farrell D, Moran J	Group early intervention eye movement desensitization and reprocessing therapy as a video-conference psychotherapy with frontline/emergency workers in response to the COVID-19 pandemic in the treatment of post-traumatic stress disorder and moral injury—an RCT study	*Frontiers in Psychology*	Original research	July 2020 to March 2022	2023	United Kingdom

Superscripted numbers refer to reference number.

**Table 3:** Summary of findings.

**Response examples**	**Advantages/best practices**	**Disadvantages/lessons learned**
**1. Mental health consultation (telephone/video/application-based consultation)**		
• Telephone support lines during working hours • 24/7 available hotlines to provide support • An online multi-component psychological intervention for HCWs • Video conference for linking with leadership • Video conference psychotherapy for individuals and group • Video debriefing • An application designed to offer individualized resources for HCWs, catering to their specific needs and providing access to a range of supportive materials and tools	• Accessible to all HCWs without any specific occupation or role • Video conference with the management team and guest speakers was reported as effective in addressing concerns, acknowledging staff, exchanging information, and providing updates • Easily duplicatable and can help all HCWs to relieve early symptoms of anxiety • Necessary steps for implementation are an early, clear mandate, adequate human resources, a functional platform (ensure anonymity), and a communication plan (send regular reminders about the hotline) • Immediate support hotline addressed some of the mild symptoms of anxiety and referred to necessary services if needed • Some hotlines were able to leave voice messages to consultants whenever • Ability to reach a greater number of participants, meet the broad mental health needs that have been detected in COVID-19, reduce future costs, and user-friendly platform regardless of internet expertise • For the online program, consistent monitoring of members’ mental health and offered weekly psychological first aid support groups for HCWs • Suggested video group therapy intervention to be reported as effective, helpful, and timely. The treatment given through video-conference platforms potentially increased availability and access • For video conferences, facilitators created a safe environment and involved members who have the same roles • Having a dedicated safe space to discuss the issues was helpful for HCWs. Debriefing was set in a way where a member could discuss with the members of the same role group, and facilitators of the sessions were familiar, trusted colleagues. The Zoom platform was easy to use • A web-based and mobile application provides advice and recommendations along with reference sources and self-assessment to be accessed in 3 different languages, even offline • Application-based mindfulness intervention was identified as effective for CBT	• Potential constraints to access the consultation services by HCWs, especially during high-intensity work regimes such as pandemics • Requires human resources in the long run • Cannot fully evaluate the effectiveness of the hotlines since no caller feedback or satisfaction were recorded • Some services did not refer to any further services or resources • Limitations in diagnosis and identification of symptoms in participants • Cannot fix problems of people with serious mental illness • Video conference and debriefing program groups might be effective only for those who are able and willing to share their issues and vulnerability with their peers and colleagues • This intervention might be efficient only for those who were comfortable sharing with peers and showing vulnerabilities, also able to use the Zoom platform • Resource contents are very broad, lacking a tailored approach for specific groups or target populations • Psychosocial interventions to have scientific and research approaches for more robust evidence on effectiveness

(Continued)

**Table 3:** Continued

**Response examples**	**Advantages/best practices**	**Disadvantages/lessons learned**
**2. Group activities/peer support**		
• Therapy sessions offered in person or virtually to HCWs • Psychological peer groups facilitated by professionals (psychologists or social workers) • Trained peers provided counseling and linked to the necessary sources • Activities determined based on the employee survey and discussion • Resilience coaching provides tailored activities for specific needs	• Using the service was therapeutic • An alternative for information dissemination and service availability for those who do not have regular access to their email • One-on-one sessions offer more in-depth support on a personal level • The peer group worked well in a setting where the professional psychological therapy sessions were stigmatized. HCWs found the peer group activities (mindfulness exercises, yoga, prayer teams) perceived as effective and tailored to their needs • Based on the actual needs of the team members, discuss the challenges openly, thus leading to more effective intervention • Staff reported that knowing about the availability of such a service was helpful • Facilitated by professionals; alleviates quick stresses and mild symptoms by discussing with other people with similar concerns • Perceived as effective in identifying and recognizing colleagues in distress, supporting and referring to the necessary sources • Addressed the staff distress caused by external and internal stresses and suggested organizational culture changes for staff wellbeing • Automated feedback on psychological characteristics has a positive effect on emotional exhaustion and does not require too much human resources • Fostered connection facilitated a safe space for emotional shifts which encouraged the expression of concerns or experiences, in addition to mental health support with mindfulness activity and learning about maintaining well-being, as well as work-life balance	• Finding available time was difficult due to overwork • Teleworking staff were not able to access • Virtual session was not acceptable to some groups • Cannot address problems for people with serious symptoms. Need to have experienced professionals to identify and refer those with serious symptoms to one-on-one sessions • In addition to the stigma associated with using therapy sessions, concerns around organizational culture, particularly confidentiality, and skepticism as the peers were providing support • Engaging as many people as possible in the co-creation of activities was a challenge. It was mentioned that the sustainability of such activities was directly linked to a leader in charge • Limited ability to overcome systemic and societal stressors
**3. Educational package (training and information support)**		
• Information dissemination through handouts, brochures, and word of mouth • Programs and resources for stress management techniques • Training for colleagues as peer supporters • Psychosocial e-learning package including various themes to address anxiety and burnout • Concise and evidence-based psycho-educational videos tailored to HCWs’ needs on various components	• The information dissemination during the in-person consultation was helpful in not missing it in the email traffic or for those who do not have regular access to their email • The information sources were available on the intranet for all staff to access during or after work hours • Colleagues/managers are trained on identifying the needs of their peers and providing support and referral to resources, which were impactful • Accessible to anybody (was accessed from low-resource countries as well as already available content) • Remotely accessible education package for all HCWs, including administrative workers, addressed self-care, psychoeducation on burnout, and preventive measures based on cultivating healthy habits	• Teleworking staff were not able to access the service • Barriers due to insufficient time in using the service • Periodical app update is needed of which the cost will be managed by the management; depending on the content some workers also reported that some videos could be shorter (20-30 min is adequate) • It can be too broad since not tailored to a specific population or occupation. Therefore, those seeking one-on-one support might not find it useful • Some video content was too long for the HCWs to finish watching in 1 sitting

(Continued)

**Table 3:** Continued

**Response examples**	**Advantages/best practices**	**Disadvantages/lessons learned**
**4. Improved environment/basic needs (therapy & rehabilitation)**		
• Comprehensive wellness practices tailored to staff needs • Psychological support through relaxation space and complementary services such as snacks, massage chairs, and therapy sessions • The wellbeing center offered donations and well-being buddies for psychological support • Hospital-led stress mitigation efforts created by the staff • Additional allowance support and promotions, as well as accommodation, transportation, meals, and logistics support for the front-line workers	• Workplace wellness program and interventions were identified as effective in preventing staff burnout and turnover, connecting others, localizing wellness activity, as well as organizational culture change • Implementation cost was relatively low • Brief breaks with accessible amenities in a quality rest space supported the emotional status of the staff • Peer support in addition to the available relaxation areas and amenities had positive effects • Perceived helpfulness and high use were mostly accessed by those who had the most needs • Team-led creative activities were tailored to needs • Extra bonus policy, reassurance from the management, understanding and communication from the leaders were useful • Various services such as basic needs and communications provided to support everyone working at the hospital in addition to the psychological support resources • Interventions focused on groups and organizational culture were perceived more positively rather than individual support measures	• Limited capacity for participation due to staff workload and lack of time • Specific groups such as younger, single, and with shorter work experience were more likely to access the service • Sustainability of the services for long-term operations was a major concern as it was costly and demanded human resources • Evidence-based approaches for the comprehensive mental health and well-being support are important for further improvement and sustainability
**5. Others**		
• Online survey • Complex measures include cognitive unloading, organizational shifts, psychoeducation training, peer counseling, well-being support, and bedside learning coordination • Enterprise initiative provides various resources in support of staff well-being through laughter yoga, health coaching, physical activity, massage therapy, nutrition, resiliency skills, etc • Breath-centered mind–body medicine program offered preventive measures in tolerating stress, maintaining an individual’s well-being, and psychosocial state • A multi-prolonged approach was implemented to mitigate burnout using the Synergy tool • Yoga and mindfulness aimed at alleviating stress and risk of burnout • Mindfulness-based intervention (MBI) facilitated by professionals online	• An anonymous online survey with an interchangeable frequency based on the needs was reported as effective in linking with leadership for rapid communication and feedback • All staff benefited from the complex measures in one way or another with the support of the dedicated team and designated director for well-being • Not only the employees benefited, but also retirees and, volunteers used the incentivized membership discounts for the program • Can be tailored to the needs of different settings because the format is simple. Themes include healthy lifestyle experiences, such as health coaching, physical activity mindfulness and meditation, massage therapy, nutrition, and resiliency skills • Daily meetings with guidance and supervision addressed the concerns immediately regarding stress, trauma, and stress-related physical conditions • Synergy tool enabled a sense of control over the workload while informing the staffing decisions based on capacity and competencies • Yoga and mindfulness have the potential to enhance self-compassion among the HCWs • Being mindful, present, and attentive to one’s own needs and emotions helped health care providers (HCPs) become calmer, more self-assured, and more connected. Their mindset shifted from an automatic, thoughtless reaction to a more mindful ability to respond to situations • The PhotoVoice technique was used to capture emotions and experiences that may be difficult to express in words. This approach significantly contributed to fostering mindfulness by encouraging individuals to articulate and share their experiences during challenging and overwhelming situations. The technique allowed them to effectively express and communicate their experiences	• More scientific and academic approaches would support health care academics • Cost was a potential barrier to healthy food besides the availability within the facility • The sessions benefited only those who were interested in learning mind–body practices • There were no follow-up data available for evaluation • Frequency of the weekly program might have been inadequate to measure the significant improvement • Not able to include nurses in the intervention as they were frontline workers during the pandemic

Abbreviations: CBT, cognitive behavioral therapy; HCW, health care worker.

### 3.2. Group activities and peer support

Group activities and peer support interventions were a different but complementary approach to providing mental health support to HCWs. These interventions allocated critical mental health resources to HCWs on the frontlines, including onsite therapy sessions,^12^^,^^17^ psychological peer group facilitation by professionals,^26-28^ counseling by trained peer supporters,^27^^,^^29^ and specific activities based on the employee surveys and discussions.^27^

#### 3.2.1. Advantages and best practices

One-on-one sessions provided more in-depth and personalized support than online consultations, and more effectively addressed the specific needs of HCWs.^26^^,^^27^ These sessions were instrumental in identifying colleagues in distress and in referring them to the most appropriate resources, thereby providing rapid and tailored support.^27^^,^^29^

Peer group activities, such as mindfulness exercises, yoga, and prayer teams, were perceived as effective in settings where there was stigma associated with the use of professional psychotherapy sessions.^26^ These activities were tailored to meet the specific needs of HCWs and aimed to foster a supportive community environment. For example, resilience coaching and peer connections at Mount Sinai Hospital helped HCWs to feel less isolated and also supported an emotional shift toward maintaining a healthy work-life balance.^30^ Similarly, small group meditation and prayer teams within hospital teams promoted bonding and significantly decreased stress.^25^ Regular debriefing meetings within teams provided continuous mental health support, and functioned as an alternative or supplementary modality to consultations with a psychologist.^22^ Automated feedback on psychological characteristics was able to reduce emotional exhaustion and did not require extensive human resources, making it a scalable and efficient intervention.^28^

#### 3.2.2. Disadvantages and lessons learned

Despite the numerous advantages, there were also several challenges and lessons to be learned. Engaging a large number of participants in the co-creation of activities was challenging, and the sustainability of these activities often required a dedicated leader.^26^^,^^31^ Finding available time for these activities was also difficult due to the demanding work schedules of HCWs.^12^^,^^29^ Another challenge was the decreased accessibility of these support systems for teleworking staff, who were often unable to participate in onsite activities.^12^ Additionally, the stigma associated with using psychotherapy sessions persisted, and this was compounded by concerns about the organizational culture, particularly confidentiality and skepticism regarding support from peers.^26^^,^^29^ This type of support was also limited, in that it was unable to address systematic and societal stressors^30^ and was insufficient for HCWs who had serious symptoms and required experienced professionals with one-on-one sessions.^28^

### 3.3. Educational packages and resources

The educational programs that were developed to support the mental health of HCWs during the pandemic were also reported as helpful. These programs disseminated information through handouts, brochures, and word-of-mouth,^12^ and were designed to teach stress management techniques.^12^ Various psychosocial e-learning packages were designed to address anxiety and burnout.^32^ Concise and evidence-based psychoeducational videos that were tailored to the specific needs of HCWs were also developed to cover various relevant issues.^33^

Hospitals employed diverse approaches for training HCWs, with some conducting in-person sessions and others using online packages that offered resources for psychological assistance and self-care.^12^^,^^23^^,^^32^ Other studies developed brief psychoeducational videos on stress management and mindfulness exercises.^22^^,^^33^ These resources were helpful to HCWs, and also reached a broad audience because they were available through various social media.^32^^,^^34^ A comprehensive approach was employed in countries such as the United Kingdom, where the National Health Service appointed a director of well-being to coordinate the activities of staff that administered mental health care. This multifaceted strategy included training in psychological personal protective equipment (PPE); educational programs on self-reflection, anxiety management, and mindfulness; pairing of staff to monitor distress of colleagues; and provision of various accommodations for resting. Additionally, workforce support desks were established in rest areas to reduce the administrative burden on the staff.^34^

#### 3.3.1. Advantages and best practices

The in-person dissemination of information particularly was seen as effective in ensuring that HCWs did not miss important updates amidst their influx of emails and other messages. Information was also made available on the intranets that was accessible to all staff during and after work.^12^ The training of colleagues and managers to help them identify the needs of their peers and provide appropriate support and referrals was very helpful.^29^ These educational resources were made accessible to a wide audience, including those in low-resource countries.^32^ Educational packages that allowed remote access addressed self-care, psychological education regarding burnout, and measures that sought to cultivate healthy habits. These resources were made accessible to all HCWs, including administrative workers.^33^

#### 3.3.2. Disadvantages and lessons learned

Despite the overall success of many educational interventions, there were also several challenges. Teleworking HCWs often lacked access to these services, a significant limitation of this approach.^12^ Another disadvantage was that HCWs often lacked the time to use these services.^29^ There was also a need for periodic updates of the educational apps, and this led to ongoing costs that required management oversight. Some workers found the video content too broad, in that it was not tailored to specific populations or occupations, making it less useful for those who needed one-on-one support.^32^ Additionally, some HCWs reported the videos were too long to watch in a single sitting, suggesting a need for shorter and more digestible content.^33^

### 3.4. Improved environment

Beyond the critical provision of PPE and medical supplies, HCWs expressed high appreciation for enhancements to the work environment and the introduction of dedicated rest areas. One of the most notable innovations during the pandemic was the creation of “wobble rooms,” also known as calm rooms, bubble rooms, time-out rooms, rainbow rooms, and safe rooms. These spaces had features such as self-massage chairs, musical relaxation sessions, and low-level lighting, and provided a much-needed respite from the chaotic hospital environment.^35-37^

The wobble rooms served a dual purpose: they were a quiet place for HCWs to rest, and they were a social space for interaction with colleagues. These interactions were among the most effective interventions, because talking to colleagues had a positive impact on the mental health of HCWs. These wobble rooms facilitated relaxation and provided a venue for HCWs to share their experiences and offer support to each other over drinks and snacks. These spaces were most frequently accessed during and after peaks of COVID-19 cases, but their usage remained steady even after peak periods, indicating their ongoing value.^36^ In the absence of dedicated rest areas, HCWs organized small team activities to relieve stress and boost morale. These activities included yoga sessions, ice cream breaks, meditation, and prayer sessions. Such team-led initiatives were tailored to the specific needs of the group, and fostered a supportive environment within the health care setting.^25^ The frontline workers also expressed appreciation for additional support measures, such as financial bonuses, promotions, and logistical support such as accommodations, transportation, and meals.^38^

#### 3.4.1. Advantages and best practices

The reviewed studies reported that participants perceived these wellness interventions as having multifaceted advantages. Workplace wellness programs were identified as effective in preventing burnout, reducing staff turnover, fostering connections among co-workers, and promoting a positive change in the organizational culture.^31^ These interventions also had relatively low costs.^31^ Brief breaks in well-equipped rest areas supported the emotional well-being of HCWs, and the addition of peer support enhanced the positive effects of these relaxation areas.^35^

Peer support, in addition to relaxation areas and amenities, was reported to have positive effects on the mental well-being of HCWs.^36^ The HCWs perceived these peer-support interventions as helpful because they were active participants, an important consideration for those with the greatest needs.^37^ Team-led creative activities, such as yoga, meditation, and prayer groups, were tailored to meet the specific needs of different teams, and they provided a supportive and inclusive environment.^25^ Moreover, the various services that addressed basic needs and the need for communication were provided to all hospital workers, and complemented the resources used for psychological support.^39^

The frontline workers also highly appreciated the many extra support measures, such as financial bonuses, promotions, and logistical support (accommodations, transportation, and meals).^38^ These measures, combined with reassurance and communications from management, were seen as particularly useful in maintaining the morale and motivation of HCWs. They had more positive perceptions of interventions that focused on group activities and organizational culture than individual support measures, highlighting the importance of collective support in crisis situations.^40^

#### 3.4.2. Disadvantages and lessons learned

The heavy workload and lack of time often limited the ability of HCWs to participate in wellness programs and use the rest areas.^25^^,^^31^^,^^36^ Additionally, specific groups of workers, such as those who were younger, single, and with less work experience, used these services more frequently, suggesting the need to address this disparity.^35^^,^^37^ The long-term sustainability of these services was a major concern, because maintaining these initiatives required significant financial and human resources.^36^ In fact, ensuring the continuity of these programs beyond the immediate crisis period posed a challenge for many health care institutions.^38^ Furthermore, although these interventions were reported as beneficial, there was a need to use more evidence-based approaches to provide comprehensive support of the mental health and well-being of HCWs.^40^

### 3.5. Other support

Some of the other forms of support that were used to maintain well-being, increase tolerance to stress, and maintain a healthy psychosocial state in HCWs were yoga, health coaching, physical activity programs, massage therapy, and breath-centered/mind-body alternative medicine programs.^41-44^ Online surveys, which were conducted at different frequencies based on need, were considered as effective in linking HCWs with the leadership for rapid communication and feedback.^12^ PhotoVoice is a visual arts–based methodology^45^ that empowers participants by allowing them to capture moments in time through photography. PhotoVoice helps an individual to capture emotions that may be difficult to convey in words; it uses visual imagery to help with the expression of complex feelings and experiences; and it fosters mindfulness by encouraging an individual to articulate experiences, particularly during challenging and overwhelming situations. PhotoVoice can also be a powerful research tool, because it enables expression of difficult feelings from an individual’s own perspective.^46^ As a user-friendly method of data collection, PhotoVoice is increasingly popular because it allows individuals to express emotions and perceptions that text alone cannot capture.^47^

#### 3.5.1. Advantages and best practices

These other support programs offered certain benefits to current HCWs and also to retirees, volunteers, and others through incentivized membership discounts. This inclusivity helped to broaden the impact of these programs, and extend support beyond the immediate workforce.^41^ These other programs were identified to be adaptable to various settings due to their simple format, and because they focused on health coaching, physical activity, mindfulness, meditation, massage therapy, nutrition, and resilience skills. These comprehensive programs were reported to address multiple aspects of well-being, and promoted a healthy lifestyle and experiences.^41^

Daily meetings with guidance and supervision were also seen as effective in promptly addressing the concerns of HCWs that were related to stress, trauma, and stress-related physical conditions. The rapid response to these problems was crucial in managing the acute stress experienced by HCWs.^42^ Additionally, the use of “synergy tools” provided HCWs with a sense of control over the workload, so that staffing decisions were based on capacity and competencies, and this enhanced operational efficiency and reduced work-related stress.^48^

Yoga and mindfulness practices enhanced the self-compassion of HCWs.^43^ More specifically, these practices were reported as helpful for HCWs to become more mindful, live in the present moment, and attend to their own needs and emotions, allowing them to develop a calmer, more self-assured, and connected approach to work. This shift from automatic and thoughtless reactions to mindful responses improved the ability of HCWs to handle stressful situations.^44^ The PhotoVoice technique was particularly noteworthy because it captured emotions and experiences that were often difficult to express verbally. This method encouraged HCWs to articulate and share experiences during challenging and overwhelming situations, and increased mindfulness and enhanced emotional expression. Overall, the ability of HCWs to more effectively communicate and share these experiences contributed to establishment of a more supportive and empathetic work environment.^44^

#### 3.5.2. Disadvantages and lessons learned

Adopting a more scientific approach would provide more robust support and help ensure that the interventions were evidence-based and effective.^34^ Additionally, the cost of healthy food and its availability within some facilities were potential barriers, and this limited the value of nutrition support programs.^41^ The support sessions primarily benefited HCWs who were already interested in mind-body practices, indicating the need to encourage all HCWs to participate in these programs.^42^ Moreover, there were no follow-up data available for evaluation, so the long-term effectiveness and impact of the interventions are unknown.^48^ Weekly programs might not yield significant improvements in well-being, suggesting a need to consider more consistent and frequent interventions.^43^ Another limitation is that nurses were not included in some interventions, due to the urgent need for their frontline services during the pandemic. This underscores the need to develop more flexible and inclusive programs that can accommodate the demanding schedules of all HCWs, particularly those on the frontlines.^44^

## 4. Discussion

This study examined the strategies from various global health care institutions that aimed to support the mental health of HCWs during the COVID-19 pandemic by focusing on best practices, advantages, lessons learned, and potential disadvantages.

Although many interventions prioritized psychological support and resilience-building for individuals, they often overlooked systemic factors contributing to HCWs’ mental health. Such a narrow focus risks perpetuating burnout by encouraging HCWs to “push through” systemic shortcomings instead of advocating for organizational reforms.^49^ Although one-time or short-term interventions may provide temporary relief, a comprehensive, system-wide approach is necessary to ensure sustainable improvements in HCWs’ well-being.^50^ To address these systemic gaps, integrating the concept of Psychosocial Safety Climate (PSC) might be a promising strategy. Over the past decade, PSC has gained recognition as a key organizational factor, primarily shaped by management, that predicts employee health outcomes.^51^ PSC refers to the shared perception among employees that their organization’s policies, practices, and procedures prioritize their psychological health and safety.^52^ Incorporating PSC into mental health interventions acknowledges that individual resilience alone cannot compensate for system-level deficits, such as understaffing, inadequate resources, and punitive workplace cultures.^53^ Therefore, we discuss how incorporating PSC can promote more sustainable and impactful mental health support for HCWs, drawing on the findings of our review.

The most common interventions identified were psychological support programs, such as counseling, therapy, and support groups, delivered through in-person sessions, hotlines, virtual platforms, and self-guided online resources. Although participants generally reported these services as beneficial, the perceived effectiveness of these interventions was limited by low participation rates,^12^^,^^13^^,^^19^^,^^22^^,^^28^ and lack of accommodation for specialty groups.^23^^,^^27^ Adopting a PSC lens would encourage health care organizations to formalize policies that ensure these programs are integrated into regular work routines and are backed by leadership commitment.^54^ For example, offering protected time to attend counseling sessions, mitigating stigma around seeking help, and actively involving managers in promoting mental health resources are essential.^55^ Co-designing services with HCWs fosters buy-in, autonomy, and meaningful engagement.^7^

Many institutions introduced educational resources, including stress management techniques, psychoeducational videos, and broader mental health programs. The immediate and practical nature of these resources helped HCWs manage acute stress. These were practical for managing acute stress but needed ongoing refinement to stay relevant. A high-PSC environment would include continuous feedback loops so that HCWs can share evolving challenges, allowing leadership to adapt or update content. This transforms these resources from one-time interventions into ongoing parts of a culture that truly values mental well-being.^52^

Some institutions created “wobble rooms” or peer support programs to offer immediate physical and emotional relief. These initiatives helped HCWs decompress and share experiences but could be expensive to maintain.^36^ A PSC-based framework urges institutions to consider not only the initial implementation but also the long-term maintenance of such interventions.^56^ Embedding wellness activities within organizational policies—such as scheduled breaks, adequate staffing, and managerial accountability—reduces the reliance on HCWs’ personal capacity to engage with these spaces and ensures their sustainability.^57^

Despite the range of interventions identified, our review highlighted that many failed to address the root causes of occupational stress. These root causes included increased job demands and work pressure,^58^^,^^59^ fear of contracting COVID-19,^60^^,^^61^ inadequate PPE,^62^ social isolation and lack of support,^58^^,^^60^ rotating shifts, long working hours,^58^^,^^62^ and insufficient training.^58^ Failing to address these factors places undue responsibility on HCWs to adapt individually. A positive PSC shows that leadership is addressing systemic stressors through policy changes, resource allocation, and training, rather than expecting HCWs to cope with unsustainable conditions.^53^ Institutions that adopt and maintain a high PSC focus on primary prevention by addressing stressors at their root, instead of relying heavily on secondary or tertiary interventions that emphasize individual coping.^52^ In addition, by meeting basic needs such as providing PPE, childcare, and rest spaces, organizations build trust and demonstrate genuine commitment to their workforce.^13^^,^^63^

Although most reviewed studies reported that interventions were effective, their overall applicability remains uncertain, as various factors can influence how interventions are perceived. For instance, cultural factors likely play a significant role in shaping perceptions of these interventions.^64^ Therefore, it is crucial to account for these variables when evaluating and implementing mental health support programs for HCWs.

Previous viral outbreaks, such as the severe acute respiratory syndrome (SARS) outbreak of 2002 to 2004, had long-term mental health consequences for HCWs.^65^ However, no COVID-19 studies have yet addressed these long-term impacts. By prioritizing PSC, health care systems can move from short-term, reactive strategies to a comprehensive, long-term commitment to HCW mental health.^53^ For example, appointing dedicated well-being directors or committees, as seen at Nightingale Hospital London in our review,^34^ exemplifies a system-level approach. Such teams help to institutionalize PSC by providing ongoing leadership focus on mental health, facilitating rapid decision-making and resource allocation during crises, and establishing protocols that remain in place post-crisis for continued support.

This scoping review has several strengths and limitations worth noting. A major strength is that we identified programs that provided evaluations, addressing a gap left by previous reviews and studies.^6^ Additionally, it highlighted best practices from many diverse initiatives, which recent Cochrane reviews did not analyze in terms of the efficacy of different strategies.^66^

However, there are also several limitations to consider. First, we focused solely on academic literature, as searching the gray literature typically requires multiple sources and strategies, particularly for non-English materials, which would have been highly time-consuming and resource-intensive.^67^^,^^68^ Second, it is important to acknowledge that our review period was from 2020 to August 2023. Consequently, we did not include developments and information published after this time. Third, most of the included papers were from high-income or upper-middle-income countries, and this limited our ability to identify strategies or programs in low-resource settings.

The findings in the reviewed studies primarily relied on participant-reported outcomes, with limited use of objective measures to assess the impact of interventions. Without incorporating objective measures, such as validated psychological scales, biological stress indicators like cortisol,^69^ or professional metrics like absenteeism and turnover,^70^ it is challenging to accurately assess an intervention’s effectiveness. To address this, future studies should combine subjective and objective data, ensuring that interventions align with perceived needs while delivering tangible, measurable benefits.

Additionally, we propose several directions for future research. First, researchers should systematically evaluate the impact of mental health interventions on HCWs, focusing on identifying which PSC-based elements remain effective over time. Second, further research is needed in lower-resource settings to understand how PSC principles can be adapted cost-effectively.

In terms of policy implication, developing clear guidelines for maintaining and scaling mental health programs is essential, including embedding PSC within Health-EDRM frameworks. Moreover, health care systems should move beyond viewing interventions as “add-ons” and instead institutionalize a PSC-driven culture by recalibrating workloads, ensuring adequate staffing, and making resources widely accessible.

## 5. Conclusion

Our review revealed a range of interventions—spanning individual counseling, wellness centers, educational resources, and organizational policies—implemented during the COVID-19 pandemic to support HCWs’ mental health. Although many were beneficial, they often emphasized individual resilience without sufficiently addressing system-level shortcomings. There is a clear need for broader and more systematic interventions that offer benefits to all HCWs, and that provide unconditional support during health care emergencies. Integrating PSC into health care organizations offers a pathway to rectify this imbalance, shifting the focus from merely supporting individual HCWs to reshaping organizational policies and practices that safeguard their well-being. By embedding PSC principles into daily operations, leadership structures, and future preparedness plans, health care institutions can better foster long-term mental health for HCWs ensuring that during crises and beyond, frontline professionals are supported not just to survive, but to thrive. In addition, comprehensive all-hazard emergency planning and disaster management are imperative to prevent burnout and address changing needs during a pandemic.

## Supplementary Material

Web_Material_uiaf020

## Data Availability

Detailed information on the reviewed articles listed in Table 1 and all articles can be found in the databases Medline, Web of Science, and Scopus.
