# Supplementary material for: Impact of the COVID-19 pandemic on health emergency and disaster risk management systems: a scoping review of mental health support provided to health care workers
Source: J Occup Health. 2025 Mar 31;67(1):uiaf020. doi: 10.1093/joccuh/uiaf020 (PMC12045710; doi:10.1093/joccuh/uiaf020)
Supplement: Web_Material_uiaf020 [file web_material_uiaf020.zip › 250121_Supplementary Tables.pdf]

**Supplementary Table 1.** Search strategy

| Database | PCC | Search term | | # of articles retrieved |
|---|---|---|---|---|
| **PubMed** | **P- Population** | #1 | ( "Health Workforce/organization and administration"[Mesh] OR "Health Workforce/statistics and numerical data"[Mesh] OR "Health Workforce/supply and distribution"[Mesh] ) | 4,551 |
| | | #2 | ( "Health Resources/organization and administration"[Mesh] OR "Health Resources/statistics and numerical data"[Mesh] OR "Health Resources/supply and distribution"[Mesh] ) | 13,623 |
| | | #3 | "Health Personnel/analysis"[Mesh] OR "Health Personnel/epidemiology"[Mesh] OR "Health Personnel/legislation and jurisprudence"[Mesh] OR "Health Personnel/organization and administration"[Mesh] OR "Health Personnel/standards"[Mesh] OR "Health Personnel/statistics and numerical data"[Mesh] OR "Health Personnel/supply and distribution"[Mesh] ) | 153,760 |
| | | #4 | ( "Allied Health Personnel/organization and administration"[Mesh] OR "Allied Health Personnel/psychology"[Mesh] OR "Allied Health Personnel/statistics and numerical data"[Mesh] OR "Allied Health Personnel/supply and distribution"[Mesh] ) | 18,456 |
| | | #5 | #1 OR #2 OR #3 OR #4 | 168,768 |
| | **C- Concept** | #6 | ( "Burnout, Professional/analysis"[Mesh] OR "Burnout, Professional/epidemiology"[Mesh] OR "Burnout, Professional/organization and administration"[Mesh] OR "Burnout, Professional/prevention and control"[Mesh] OR "Burnout, Professional/psychology"[Mesh] OR "Burnout, Professional/statistics and numerical data"[Mesh] )) OR ( "Burnout, Psychological/analysis"[Mesh] | 12,802 |
| | | #7 | "Burnout, Psychological/epidemiology"[Mesh] OR "Burnout, Psychological/organization and administration"[Mesh] OR "Burnout, Psychological/prevention and control"[Mesh] OR "Burnout, Psychological/psychology"[Mesh] OR "Burnout, Psychological/statistics and numerical data"[Mesh] )) | 13,038 |
| | | #8 | ( "Mental Health/epidemiology"[Mesh] OR "Mental Health/organization and administration"[Mesh] OR "Mental Health/prevention and control"[Mesh] OR "Mental Health/statistics and numerical data"[Mesh] )) | 6,516 |
| | | #9 | ( "Mental Health Services/methods"[Mesh] OR "Mental Health Services/organization and administration"[Mesh] OR "Mental Health Services/statistics and numerical data"[Mesh] OR "Mental Health Services/supply and distribution"[Mesh] ) | 47,181 |
| | | #10 | ( "Stress, Psychological/epidemiology"[Mesh] OR "Stress, Psychological/etiology"[Mesh] OR "Stress, Psychological/organization and administration"[Mesh] OR "Stress, Psychological/prevention and control"[Mesh] OR "Stress, Psychological/statistics and numerical data"[Mesh] ) | 65,363 |
| | | #11 | ( "Stress, Physiological/epidemiology"[Mesh] OR "Stress, Physiological/etiology"[Mesh] OR "Stress, Physiological/organization and administration"[Mesh] OR "Stress, Physiological/prevention and control"[Mesh] OR "Stress, Physiological/psychology"[Mesh] OR "Stress, Physiological/statistics and numerical data"[Mesh] ) | 65,363 |
| | | #12 | ( "Occupational Stress/epidemiology"[Mesh] OR "Occupational Stress/etiology"[Mesh] OR "Occupational Stress/organization and administration"[Mesh] OR "Occupational Stress/prevention and control"[Mesh] OR "Occupational Stress/statistics and numerical data"[Mesh] ) | 11,505 |
| | | #13 | ( "Anxiety/epidemiology"[Mesh] OR "Anxiety/etiology"[Mesh] OR "Anxiety/organization and administration"[Mesh] OR "Anxiety/prevention and control"[Mesh] OR "Anxiety/psychology"[Mesh] OR "Anxiety/statistics and numerical data"[Mesh] ) | 67,902 |
| | | #14 | ( "Depressive Disorder/epidemiology"[Mesh] OR "Depressive Disorder/organization and administration"[Mesh] OR "Depressive Disorder/prevention and control"[Mesh] OR "Depressive | 63,145 |

| | | | | |
|---|---|---|---|---|
| | | | Disorder/psychology"[Mesh] OR "Depressive Disorder/statistics and numerical data"[Mesh] ) | |
| | | #15 | "Psychosocial Support Systems"[Mesh] | 1,001 |
| | | #16 | #6 OR #7 OR #8 OR #9 OR #10 OR #11 OR #12 OR #13 OR #14 OR #15 | 272,989 |
| | **C-Context** | #17 | (( "COVID-19/analysis"[Mesh] OR "COVID-19/epidemiology"[Mesh] OR "COVID-19/organization and administration"[Mesh] OR "COVID-19/prevention and control"[Mesh] OR "COVID-19/psychology"[Mesh] OR "COVID-19/statistics and numerical data"[Mesh] )) | 84,984 |
| | | Combine | #5 AND #16 AND #17 | 218 |
| | | #18 Limit | **Limit to English Language** | **214** |

| | | |
|---|---|---|
| Scopus | ( TITLE-ABS-KEY ( covid-19 ) AND TITLE-ABS-KEY ( "Health* worker*" OR "Frontline worker*" ) AND TITLE-ABS-KEY ( burnout OR "occupational safety" OR "occupational stress" OR "psychosocial support" ) ) AND ( LIMIT-TO ( OA , "all" ) ) AND ( LIMIT-TO ( SUBJAREA , "MEDI" ) OR LIMIT-TO ( SUBJAREA , "NURS" ) OR LIMIT-TO ( SUBJAREA , "HEAL" ) ) AND ( LIMIT-TO ( LANGUAGE , "English" ) | **811** |

| | | |
|---|---|---|
| Web of Science | ((TS=(COVID-19)) AND TS=("Health* worker*" OR "Frontline worker*" )) AND TS=(burnout OR "occupational safety" OR "occupational stress" OR "psychosocial support") and English (Languages) and Nursing or Health Care Sciences Services or Public Environmental Occupational Health or Psychology (Research Areas) and Open Access and Public Environmental Occupational Health or Health Care Sciences Services or Health Policy Services or Nursing or Psychology Multidisciplinary or Psychology Clinical or Industrial Relations Labor or Psychology | **305** |

Search strategy was conducted in accordance with PCC – Participant, Concept, Context Framework.

Participant - Healthcare workers
Concept - Physiological and psychological burnout, mental disorders including stress, anxiety, depression
Context - COVID-19

Publication date: From 2020-01-01 to 2023-09-30
Search date: 2023-10-06

**Supplementary Table 2.** Responses on mental health services provided with healthcare workers and their best practices and lessons to be learned described in reviewed articles

| N | Article information | Responses | Types of responses | Study setting | Perceived effectiveness of response | Advantage/Best practice | Disadvantage/Lessons to be learned |
|---|---|---|---|---|---|---|---|
| 1 | Hoedl M, Osmancevic S, Thonhofer N, Reiter L, Schoberer D. Psychosocial interventions for healthcare workers during the COVID-19 pandemic: rapid review and meta-analysis. 2024;174(3-4):79-86. | A psychoeducational app called PsyCovidApp, created by mental health experts. The app content was based on aspects of cognitive-behavioral therapy and mindfulness. The intervention group had access to the app for 14 days, during which they received daily notifications or were asked to answer short 25:25 that had been designed to encourage improvements in lifestyle, stress, social support, or emotional skills.<br><br>Mindfulness-based stress reduction - For this intervention, the intervention group received audio and video files with instructions for performing meditation and yoga exercises. The group also received texts on the benefits of mindfulness, and audio and video files with mind exercises guided by professionals.<br><br>Nurses were provided with psychological counselling. As psychological support, participants received counselling and instruction on mindfulness decompression therapy from psychologists to encourage mindfulnes. | Mental health consultation | Hospitals<br><br>(Hospitals and long-term care institutions) | NA.<br>No robust evidence was found that psychosocial interventions can reduce psychological distress. No significant effect could be found regarding post-traumatic stress. The psychosocial intervention had no beneficial effect on burnout and depression, whereas providing a mindfulness- or relaxation-based intervention resulted in a significant improvement in sleep quality. | That mindfulness interventions can be significantly effective, and these types of interventions may be beneficial for HCW. | Interventions that are considered as psychological interventions must be clearly defined to execute targeted actions to protect HCWs from further negative psychological outcomes. |
| 2 | McLean CP, Betsworth D, Bihday C, et al. Helping the Helpers: Adaptation and Evaluation of Stress First Aid for Healthcare Workers in the Veterans Health Administration During the COVID-19 Pandemic. Workplace Health Saf. 2023;71(4):162-171. | Stress First Aid -self-care and coworker support program - | Group activities/peer support | Healthcare system<br><br>VA Connecticut Healthcare System<br><br>Iowa City VA Health Care System<br><br>Minneapolis VA Health Care System | Positive<br>The SFA program was well-received by participants. The program have workgroups a shared language to discuss stress reactions and helped shift team norms around stress disclosure. It also helped them to feel more aware of their stress reactions and empowered to care care themselves and support their peers.<br><br>Delivering a brief group SFA program targeting self-care and peer support appears to | The program was valued for its ability to (a) create a shared language to discuss stress reactions, (b) shift team norms around stress awareness and disclosure, and (c) empower staff to care for themselves and support their peers.<br><br>An important component of this program was the group format, which allowed for sharing of ideas and modeling of peer support. Participants | SFA would have been more impactful if it had been in place prior to the start of the pandemic.<br><br>It was insufficient and plans to sustain positive effects of the program differed across groups (e.g., training an in-house SFA champion, planning self-guided SFA booster sessions).<br><br>The protected time for HCW to participate in SFA whenever possible - This was a practical concern, as staff were sometimes called away for patient care during the brief sessions. In addition, not scheduling during lunch |

| | | | | | | | |
|---|---|---|---|---|---|---|---|
| | | | | New Jersey VA Health Care System<br><br>VISN 19 Clinical Resource Hub | be feasible and acceptable to hospital staff. | felt more aware of the impact of stress and how to manage it, and that the program normalized experiencing stress.<br><br>Participants felt that SFA gave their team a shared language to talk to each other about stress and permission to check in on each other regularly. This suggests that the SFA program was successful in helping to shift workplace norms about discussion and stress management.<br><br>It also appeared to be successful in meeting an immediate need for emotional support among HCW faced with significant stressors at work and at home during a pandemic. | breaks or immediately prior to the end of the work week is important.<br><br>Lessons learnt:<br>Making program participation voluntary both in terms of attending the sessions as well as participating (e.g., turning on their camera, sharing via chat or speaker) to promote psychological safety<br><br>Value of implementing a brief supervisor needs assessment to understand the group's dynamics and any major stressors impacting the team prior to initiating SFA. Facilitators hypothesize that performing a supervisor needs assessment, reaffirming psychological safety and the group's collective strengths throughout, and using a more flexible model of SFA helped to increase in group engagement. |
| 3 | Simms L, Ottman KE, Griffith JL, et al. Psychosocial Peer Support to Address Mental Health and Burnout of Health Care Workers Affected by COVID-19: A Qualitative Evaluation. Int J Environ Res Public Health. 2023;20(5):4536. | Peer caregiver (Care-for caregiver program) | Group activities/peer support<br><br>Educational package (Training and Information support) | University health care system | Positive<br>The psychosocial peer support programs for health care workers are feasible and potentially impactful but also require other systemic changes within a health care system to improve and sustain staff well-being. The training would help them to better identify their colleagues in distress, improved their listening skills, improved their ability to communicate, gave them tips about the dos and don'ts of providing peer counseling, and made them feel more confident in having difficult conversations. | Care for Caregivers program implemented at a university health care system helped to shift the organizational culture, taught staff skills for recognizing and supporting distress, and supported the staff who were already providing these services. | Raising staff awareness was the most commonly raised suggestion.<br><br>Other suggestions included on the format (making the groups between 4-15, on the length and structure (making cheat-sheets, short videos).<br><br>Another theme was the importance of training. Ideas for new audiences that should be trained as Peer Caregivers included medical students, residents, and making it available to all staff in both management and not in management roles.<br><br>Participants discussed the need to carefully assign staff to Peer Caregivers to address hierarchical and confidentiality concerns.<br><br>A few participants highlighted the importance of ongoing support and training, after the initial Peer Caregiver and Manager training courses. |

| | | | | | | | |
|---|---|---|---|---|---|---|---|
| 4 | Chandler AB, Wank AA, Vanuk JR, et al. Implementing Psychological First Aid for Healthcare Workers During the COVID-19 Pandemic: A Feasibility Study of the ICARE Model. J Clin Psychol Med Settings. 2023;30(3):482-489. | Psychological first aid (PFA)- online program to address the psychological support needs of HCWs . | Mental health consultation | Healthcare facilities in Southern Arizona | Positive<br>The implementation of an online PFA program was found to be feasible. Also, the HCW HOSTED ICARE program is believed to have the capability of being adapted to dynamic conditions. | During onboarding phone calls, symptom monitoring check-ins, and via email communication that it was helpful to know.<br><br>Support was available if they needed it.<br><br>Consistent monitoring of members' mental health and offered weekly psychological first aid support groups for HCWs. | The relatively limited utilization of online PFA support groups (0-2 members per group session). Low attendance may have been due to the structure and context of the program. HCWs were eligible to participate in the ICARE groups, which were led by non-HCWs (i.e., advanced clinical psychology graduate students), following enrollment in HCW HOSTED, an organization outside of their workplace. Peer-based support groups, either in-person or on a video platform, that are integrated within the healthcare setting could provide a better structure for HCWs in terms of program awareness and ease of accessibility (see Blake et al., 2021; Kroll et al., 2021; Malik et al., 2021). |
| 5 | Farrell D, Moran J, Zat Z, et al. Group early intervention eye movement desensitization and reprocessing therapy as a video-conference psychotherapy with frontline/emergency workers in response to the COVID-19 pandemic in the treatment of post-traumatic stress disorder and moral injury-An RCT study. Front Psychol. 2023;14:1129912. | Group intervention - early intervention, group trauma treatment, delivered remotely as video-conference psychotherapy (VCP). This early intervention used an intensive treatment delivery of 4x2h sessions over 1-week. | Mental health consultation | The Republic of Ireland and internationally from *the United States, Canada, Australia, New Zealand, Greece, and Turkey* | Positive<br>The VGTEP treatment intervention was effective for both groups, as well as suggested helpfulness and timeliness. | Video Group Therapy intervention was effective, helpful, and timely. The treatment given through video-conference platforms potentially increased availability and access. | The interventions was conducted within only one week, further research needs to consider this period.<br><br>Future early intervention should consider comparing VGTEP with another Trauma-focused CBT Group Intervention. |
| 6 | Gerbarg PL, Dickson F, Conte VA, Brown RP. Breath-centered virtual mind-body medicine reduces COVID-related stress in women healthcare workers of the Regional Integrated Support for Education in Northern Ireland: a single group study. Front Psychiatry. 2023;14:1199819. | Breath-Body-Mind Introductory Course (BBMIC), a breath-centered Mind-body Medicine program (12 h manualized training provided live online 4 h/day for 3 consecutive days followed by 6 weeks of once-a-week online group practice (45 min per session) and daily home practice (recommended 20min per day of coherent breathing with some movement practices)). | Others | 5 Health and Social Care Trust of the Regional Integrated Support for Education, Northern Ireland | Positive.<br>Participation in the BBMIC significantly reduced scores for Perceived Stress, Stress Overloads, and Exhaustion. EFI Revitilization and Tranquility scores significantly improved.<br><br>More than 60% of participants reported moderate to very strong improvements in 22 indicators of psychophysiological state, e.g., tension, mood, sleep, mental focus, anger, connectedness, | The use of manualized, previously tested BBM interventions taught by experienced faculty.<br><br>The use of codes to preserve participant confidentiality and blinding of those who collected and analyzed the data.<br><br>The faculty were trained and led by who have taught BBM for over 15 | |

| | | | | | | | |
|---|---|---|---|---|---|---|---|
| | | | | | awareness, hopefulness, and empathy. | years to disaster survivors and others with stress, trauma, and stress-related physical conditions.<br><br>Daily meetings enabled the teachers to discuss the participants and receive guidance and supervision from the faculty. Thus, problems could be addressed quickly.<br><br>It is possible to prescribe specific breath practices that integrate easily into treatments for a wide range of mental and physical disorders, as well as for prevention and performance enhancement. The practices used for everyday stress can be used for mass disasters to support better functioning and recovery.<br>(1) No control group, (2) The selection process may have favored employees who were more motivated to learn mind-body practices. | |
| 7 | Havaei F, MacPhee M, Ma A, et al. Implementation of the Synergy Tool: A Potential Intervention to Relieve Health Care Worker Burnout. Int J Environ Res Public Health. 2022;20(1):489. | Synergy Tool - tool to buffer from the burn-out among nurses - A multi-pronged approach to mitigating burnout: (a) by addressing work environment factors associated with improved provider outcomes, such as control over practice and workload management; (b) by acknowledging and valuing providers' expertise at patient assessment, care planning and delivery; and (c) by generating rigorous assessment-driven data about patient care needs to inform staffing decisions and resource allocation. | Others | Canadian tertiary-care urban hospital | N/A<br>Post intervention survey is planned to be conducted in 2023. | It has potential to facilitate workload management and to foster a sense of control among healthcare workers.<br><br>Synergy tool facilitates workload management in two important ways (a) using individual patient acuity and dependency needs to inform staffing decisions bot in terms of staffing levels and skill mix and (b) using these patient needs to assign staff based on their competencies and | The tool has not been specifically used as a burnout mitigation strategy.<br><br>Proposal for the improvement: Adding the Synergy tool to other evidence-based high involvement work practices, given our initial successes at engaging direct healthcare providers and management in a patientcentered, solution-focused approach to workload management concerns. Synergy tool use acknowledges that direct care staff are the experts when it comes to 'fit' between staff competencies and patients' priority care needs. |

| | | | | | | | |
|---|---|---|---|---|---|---|---|
| | | | | | | capacity to meet patient needs. | |
| 8 | Miyoshi T, Ida H, Nishimura Y, Ako S, Otsuka F. Effects of Yoga and Mindfulness Programs on Self-Compassion in Medical Professionals during the COVID-19 Pandemic: An Intervention Study. Int J Environ Res Public Health. 2022;19(19):12523. | A weekly one-hour yoga and mindfulness program was implemented (3 months). | Others | Okayama University Hospital | Neutral/No effectiveness<br>In the current study, the yoga and mindfulness program did not decrease the number of people experiencing burnout and didn't affect overall self-compassion. | | Frequency of the program may have been insufficient to see positive outcome of the program. Virtual sessions may have contributed to the effectiveness of the program. |
| 9 | Osman I, Singaram V. Using PhotoVoice to understand mindfulness in health care practitioners. Health SA. 2022;27:1942. | Mindfulness based intervention (MBI) - Weekly one group sessions about mindfulness facilitated by professionals online for four weeks using PhotoVoice technique. | Others | The public health care system in South Africa | Positive<br>Being mindful, being present to their experiences and considering their own needs and emotions helped HCPs be calmer, more self-assured, and more connected. Their perspectives changed from mindless reaction to a mindful ability to respond. | Capturing emotions that words might fail to convey through visual imagery was made possible by employing the Photovoice technique. This approach significantly contributed to fostering mindfulness by encouraging individuals to express and articulate their experiences during challenging and overwhelming situations.<br><br>Participants, representing diverse backgrounds and professions, found the method to be universally effective, transcending occupational differences and personal backgrounds | |

| | | | | | | | |
|---|---|---|---|---|---|---|---|
| 10 | Martindale SL, Shura RD, Cooper MA, et al. Operational Stress Control Service: An Organizational Program to Support Health Care Worker Well-Being. J Occup Environ Med. 2022;64(1):64-70. | The Combat Operational Stress Control (COSC) program was designed to proactively identify and manage stress among service members while enhancing their resilience against stress-related injuries. This initiative incorporates various strategies such as transparent communication, employee surveys, dedicated COVID-19 telephone support lines, walkabout therapies, and employee wellness programs. These measures collectively aim to address and mitigate stress factors among service members, promoting their mental well-being and resilience in challenging operational environments. | Mental health consultation<br><br>Group activities/peer support<br><br>Educational package (Training and Information support)<br><br>Others | (Veterans Affairs Health Care System encompassing a primary medical center and three associated healthcare center facilities | Positive<br>The Operational Stress Control Service (OSCS) greatly improved communication across the medical center by deploying numerous new forms of bi-directional information exchange. The immediacy of the feedback systems allowed employees to directly see responses to their concerns. | Multidisciplinary;<br>Bottom-up information sharing empowers workers to navigate away from uncertainty.<br><br>Surveys offer detailed analyses of program outcomes, facilitating an accelerated flow of information between leadership and employees.<br><br>Establishing a centralized and easily accessible point for all COVID-19-related resources has streamlined access to crucial information and support mechanisms. | HCWs do not have the time or flexibility in their schedule to realistically attend such programs, even of they know it would be helpful and desire to do so. Only about 7% of the work force was represented in the survey. |
| 11 | Dominguez-Rodriguez A, Martínez-Arriaga RJ, Herdoiza-Arroyo PE, et al. E-Health Psychological Intervention for COVID-19 Healthcare Workers: Protocol for its Implementation and Evaluation. Int J Environ Res Public Health. 2022;19(19):12749. | Developed and implemented "Personal COVID" intervention - an online multi-component psychological intervention for HCWs | Mental health consultation | Healthcare workers in Mexico | N.A | Advantages are anonymity, ability to reach a greater number of participants, meet the broad mental health needs that have been detected in the COVID-19, could reduce future costs, user-friendly platform regardless of the internet expertise. | The platform does not allow to clearly identify the symptoms of the participants. |

| | | | | | | | |
|---|---|---|---|---|---|---|---|
| 12 | Maunder RG, Kiss A, Heeney N, et al. Randomized trial of personalized psychological feedback from a longitudinal online survey and simultaneous evaluation of randomized stepped wedge availability of in-person peer support for hospital staff during the COVID-19 pandemic. Gen Hosp Psychiatry. 2023;84:31-38. | Personalized self-awareness feedback (PSAF) and Peer Resilience Champions (the PRC intervention) - recurrent, brief personalized feedback about psychological variables buffered the impact of stressors on emotional exhaustion. | Group activities/peer support | Teaching hospital and a rehabilitation hospital of Sinai Health | Neutral.<br>A small but significant beneficial effect of PSAF over time; the difference at individual timepoints was only significant at timepoint of six months.<br><br>The study did not find conclusive evidence supporting the notion that in-person peer support reduces emotional exhaustion. However, due to limitations in survey design and evaluation methods, this research neither confirms the effectiveness of peer support nor demonstrates its ineffectiveness definitively. | The study explores the impact of frequent, automated feedback regarding individual psychological traits as a means to mitigate the effects of high occupational stress on emotional exhaustion and the cost of implementing this automated feedback system through a survey platform is relatively economical, with accessibility contingent solely upon internet availability. | As a low-cost, easy-access intervention with significant benefits on an outcome, burnout, which is common and need not indicate the presence of mental disorder, an intervention like PSAF could be considered in an early phase of a stepped care approach to supporting healthcare workers, with more intensive resources reserved for those with greater needs. |
| 13 | Korman MB, Steinberg R, Gagliardi L, et al. Implementing the STEADY Wellness Program to Support Healthcare Workers throughout the COVID-19 Pandemic. Healthcare (Basel). 2022;10(10):1830. | Established a staff wellness working group which included psychiatrists, spiritual care providers, organizational development associates, occupational health and safety staff, hospital leadership, and members of the STEADY (Social Support, Tracking Distress, Education And Discussion, community) research team. Steady program which targets evidence-informed mediators of post-traumatic stress injury and resilience in first responder and healthcare worker populations (social support, earlier intervention for distress, substance use, stigma, and burnout). The program is composed of five key components: Peer Partnering, Wellness Assessments, Psychoeducation Workshops, Peer Support Discussions (general groups and critical incident stress debriefing) and Community-Building Activities.<br><br>Provided virtual one-on-one peer support session offerings with volunteers from the Department of Psychiatry. | Group activities/peer support | Selected high-need units within Canada's largest trauma hospital | Positive<br>Feedback was virtually entirely positive. Requests for support were received from approximately twenty non-target units based on positive word-of-mouth, indicating program acceptability and success. Based on feedback received and observations over the course of programming, it was concluded that this project was highly successful. | Initially planned to be entirely virtual sessions - later adjusted to in-person/virtual sessions to be more effective. The programming was flexible - adjusting to the needs of different units (e.g some units requested 30 minutes sessions instead of 1 hour, some units needed more educational sessions instead of peer support sessions).<br><br>Sessions were run by peer-champion where available and necessary instead of STEADY facilitator. Feedback through emails and messaging revealed that participants found the sessions informative and useful. | Rates of participation was low when sessions were offered entirely virtually. Some participants expressed that they were frustrated with communal support groups - needed more individual one-on-one sessions.<br><br>Future program implementation might benefit from a prolonged planning stage wherein a larger number of the target population are surveyed or consulted to identify barriers/facilitators.<br><br>Setting more reminders to the participants in various ways can help encourage participation.<br><br>Help with connecting to zoom sessions (there might be lack in technological skills).<br><br>Having familiar facilitators (peer-champions) instead of unfamiliar outsider-facilitator.<br><br>Removing the stigma related to joining the emotional support sessions. Finding allies in the leadership will help to facilitating the program uptake. |

| | | | | | | | |
|---|---|---|---|---|---|---|---|
| 14 | Olcoń K, Allan J, Fox M, et al. A Narrative Inquiry into the Practices of Healthcare Workers' Wellness Program: The SEED Experience in New South Wales, Australia. Int J Environ Res Public Health. 2022;19(20):13204. | Implemented SEED (Stability, Encompassing, Endurance and Direction) staff-led wellbeing initiatives that build resilience and aid recovery processes. Activitties include (1) Coffee Buddies—a planned coffee break with a nominated staff member as a form of collective care for each other, (2) Quiet Room—a space inside the hospital for staff to take a moment each day for a quiet reflection, (3) 24/7 Wellness—weekly wellness sessions in the workplace to promote staff wellbeing, (4) (S)Crap book—a communal journal to share and reflect on stressful stories occurring during the pandemic. | Improved environment/basic needs (Therapy & rehabilitation) | Milton Ulladulla Hospital and other sites | Positive<br>The satisfaction of staff participating in the study was almost universal. | A key SEED practice of co-design allowed for the broad engagement of all team members including administration, security, nurses, and leaders, rather than focusing on any single group.<br><br>Responsive and compassionate leadership was pivotal as it empowered leaders to promptly address and validate staff concerns. It was the foundation of all other practices.<br><br>A co-designed program was implemented, tailored to meet the specific needs of our staff based on the premise that each site needed to develop wellness activities that fit their staff's needs, resulting in situated and tailored wellness practice approaches rather than implementing a one-size-fits-all intervention.<br><br>Creating safe spaces and integrating storytelling sessions within various activities provided opportunities for staff to express themselves and feel heard.<br><br>The SEED program was executed at a low cost. | Engaging a broad spectrum of individuals in the co-design process and ensuring its ongoing maintenance posed significant challenges.<br><br>In the healthcare setting, a prevailing culture prioritizing patient care often hindered efforts to prioritize the well-being of healthcare workers and self-care.<br><br>The sustainability of the program largely hinges on the commitment of the overseeing leader; active involvement and desire to sustain the program will dictate its continuity.<br><br>Workplace wellness interventions may be needed to not only prevent staff burnout and turnover but also to lead organizational culture change. |
| 15 | d'Ussel M, Adam F, Fels A, Chatellier G, Philippart F. Characteristics of Hospital Workers Using a Wellbeing Center Implemented During the COVID-19 Pandemic to Prevent the Emotional Impacts of the Crisis. Front | Space for both relaxation and access to necessary services and resources. Offers refreshments and sweets, massage chair, therapy sessions on reservation. | Improved environment/basic needs (Therapy & rehabilitation) | Saint-Joseph hospital; Marie Lannelongue hospital | N.A.<br>Difficulties in evaluating the effect of the well-being center on anxiety, depression, or PTSD. | Accessible for all HCWs (including administrative workers) | The center was mostly accessed by younger workers (ages 18-25, single, shorter work experience ), those who accessed psychological service were more likely to access the bulle. |

| | | | | | | | |
|---|---|---|---|---|---|---|---|
| | Public Health. 2022 Jul 4;10:913126. | | | | | | |
| 16 | Rosen B, Preisman M, Read H, et al. Resilience coaching for healthcare workers: Experiences of receiving collegial support during the COVID-19 pandemic. Gen Hosp Psychiatry. 2022;75:83-87. | Group activity: Resilience coaching was provided to team members, tailoring activities according to the specific needs of each team. This included leading mindfulness activities or engaging in discussions centered around challenges encountered by the unit. | Group activities/peer support | Mount Sinai Hospital and Hennick Bridgepoint Hospital | Positive<br>Participants indicated greatest benefits in emotion and meaning-based coping. Sessions were safe place to regulate emotions, which produced less alone feeling. Staffs acknowledged emotional shifts toward being able to maintain work-life balance. | A safe environment was created to encourage the expression of concerns and the sharing of personal experiences.<br><br>A resource center was established to provide information and support related to mental health resources. | The implementation of this service necessitates the involvement of an experienced psychiatric department. In this hospital, the Psychiatric Department boasts credible psychologists capable of managing such a service. The leadership was not directly engaged in this initiative. Resilience Coaching cannot overcome systemic and societal stressors. Male and physicians participated less in the study. |
| 17 | Kelsey EA. Joy in the Workplace: The Mayo Clinic Experience. Am J Lifestyle Med. 2021;17(3):413-417. Published 2021 Aug 6. | Wellness program: Mayo clinic devloped an enterprise initiative, called Joy at Mayo Clinic; to infuse joy into the workplace as a priority<br><br>The Well-Being Champion Program provides resources to share across the enterprise including a variety of health topics from recipes to participating in laugher yoga. | Others | The Mayo Clinic | NA.<br>Impact of the project or intervention is measured at 6 and 12 months through recommended surveys including measurements of professional burnout, the Well-Being Index (WBI), or metrics available through the All Staff Survey. | Mayo Clinic maintains a website on the employee intranet to share stories of these themes resonating across the enterprise. All employees have the opportunity to participate in Well-Being Champion Program. The Well-Being Champion Program provides resources to share across the enterprise including a variety of health topics from recipes to participating in laugher yoga.<br>Healthy lifestyle experiences, such as health coaching, physical activity mindfulness and meditation, massage therapy, nutrition, and resiliency skills, are some of the resources available.<br><br>Employee Assistance Program (EAP) is available for confidential and professional short term counseling at no cost. __the primary Mayo Clinic employee exercise facility, located at the | |

| | | | | | | | |
|---|---|---|---|---|---|---|---|
| | | | | | | DAHLC, had been shut down for greater than 12 months during the pandemic. As an employee benefit, Mayo Clinic employees, volunteers, retirees, and spouses are eligible to belong to this facility and encourage utilization by incentivized membership discounts based upon frequency of use. Some offerings, such as personal training and group fitness classes, were modified into a virtual model to accommodate fitness needs during the pandemic. | |
| 18 | Petrella AR, Hughes L, Fern LA, Monaghan L, Hannon B, Waters A, Taylor RM. Healthcare staff well-being and use of support services during COVID-19: a UK perspective. Gen Psychiatr. 2021 Jun 22;34(3):e100458. | Offered service for free food, donations and care packages, off-site respite centre | Improved environment/basic needs (Therapy & rehabilitation) | Central London hospital | Positive/Neutral<br>There was a positive relationship between service use and both exposure to morally distressing situations the exposure to morally distressing situations and higher levels of psychological distress experienced, the more engaged respondents were in support services. In addition, awareness of available services and resources was high, signifying that efforts to signpost staff to available supports were effective. | Availability of various services was advantage. Awareness of the services were also high. Those with higher burnout levels were more likely to access these services. Most commonly accessed services were the ones that were less stigmatized. | Psychosocial, welfare services were accessed the least and were more likely to be aware by HCWs. |

| | | | | | | | |
|---|---|---|---|---|---|---|---|
| 19 | Mira JJ, Cobos-Vargas Á, Astier-Peña MP, Pérez-Pérez P, Carrillo I, Guilabert M, Pérez-Jover V, Fernández-Peris C, Vicente-Ripoll MA, Silvestre-Busto C, Lorenzo-Martínez S, Martin-Delgado J, Aibar C, Aranaz J. Addressing Acute Stress among Professionals Caring for COVID-19 Patients: Lessons Learned during the First Outbreak in Spain (March-April 2020). Int J Environ Res Public Health. 2021 | The core group identified and categorized the most common problems then developed intervention suggestions with guidance from mental and occupational health experts'. These interventions include: (1) aimed to strengthening work morale, (2) aimed at better equipping isolated personnel to be of service, (3)to provide professionals with resources to deal with the emotional overload caused by caring for patients with COVID-19 and (4) to strengthen the role of middle managers. | Improved environment/basic needs (Therapy & rehabilitation) | Thirteen healthcare institutions from eight autonomous communities in Spain | N/A<br>The current state of emergency has not allowed for an empirical analysis of the effectiveness of proposed interventions. | The core group members were from diverse backgrounds including primary care area managers, psychiatrists, occupational health physicians, nurses, psychologists and pharmacist. In this case, it has been especially useful to incorporate their knowledge of the experience being lived in the centers.<br>Sharing information to gauge expectations, listening to what professionals need, feeling protected from threats, organizational flexibility, encouraging teamwork, and leadership that promotes psychological safety have led to more positive responses. | The effectiveness of each of these interventions for the purpose for which they have been applied has not been contrasted through experimental or quasi-experimental studies. |
| 20 | Collins GB, Ahluwalia N, Arrol L, et al. Lessons in cognitive unloading, skills mixing, flattened hierarchy and organisational agility from the Nightingale Hospital London during the first wave of the SARS-CoV-2 pandemic. BMJ Open Qual. 2021;10(3):e001415. | Complex measure: Cognitive unloading strategy: Director of Wellbeing was appointed alongside the committee of psychologists, occupational health specialists, human resource managers and psychiatrists.<br><br>Psychological PPE training - educational programme to educate staff in self-reflection, anxiety management and mindfulness<br><br>Paired with colleague before each shift to monitor any distress<br><br>Physical wellbeing support - food, drink, parking accomodation<br><br>Bedside Learning Coordinator - incident reporting and education of staff about protocol updates in real time | Others | Nightingale Hospital London | Positive<br>Clinical outcomes were comparable to existing clinics with larger ICU units. The Nightingale Hospital London patient mortality was 48.1% compared with 47.7% nationally, and the requirement for, and duration of, organ support were also similar. | The most complex support among all - for health care workers, each worker can benefit in one way or another.<br><br>Having dedicated team and Director of wellbeing behind can make the problem solving and decision making fast during emergencies.<br><br>The interventions offered in this study not only necessary during emergency, but also can be relevant during non-pandemic time. | |

| | | | | | | | |
|---|---|---|---|---|---|---|---|
| 21 | Norful AA, Rosenfeld A, Schroeder K, Travers JL, Aliyu S. Primary drivers and psychological manifestations of stress in frontline healthcare workforce during the initial COVID-19 outbreak in the United States. Gen Hosp Psychiatry. 2021 Mar-Apr;69:20-26. | Various initiatives provided from the hospital side HCWs created small activities within the teams such as an "all hands on deck" approach, social media support, and hospital led programs on stress mitigation. | Improved environment/basic needs (Therapy & rehabilitation) | Urban academic medical centre in Washington, DC | Positive<br>Organizational support and promotion of team response were effective for clinician well-being. Also, social media support was deemed to be effective from the perspectives of the healthcare workforce in this study. Stress mitigation efforts created by the workforce themselves, both within and outside the hospital, were viewed as more effective. | Daily email from the president were supportive. HCWs created their own activities including yoga, occasional free food within the teams, prayer teams. Since teams created these activities by themselves, these were effective and tailored to the needs. | Hospital has also offered psychosocial program, but employees did not see the need for themselves to participate. Also, the timing of the psychological services was not appropriate (not allowed during the shifts, either after or on off days) |
| 22 | Zhu Z, Xu S, Wang H, Liu Z, Wu J, Li G, Miao J, Zhang C, Yang Y, Sun W, Zhu S, Fan Y, Chen Y, Hu J, Liu J, Wang W. COVID-19 in Wuhan: Sociodemographic characteristics and hospital support measures associated with the immediate psychological impact on healthcare workers. EClinicalMedicine. 2020 Jun 24;24:100443. | Hospital-based and department-based support measures included an additional allowance for FHWs, preferential policies on the promotion of professional titles, regular greetings, and reassurance from hospital executives, nursing leaders, and department chairs for all HWs, as well as COVID-19 infection was treated as a work-related injury. Accommodation was offered. Transportation and food were prepared for the HCWs. Full-coverage protective measures were disinfection of the workplace, shutting of central air-conditioning, and dissemination of facts on COVID-19 prevention. | Improved environment/basic needs (Therapy & rehabilitation) | Tongji Hospital | Positive<br>91.0% of HCWs reported they had received hospital-based and department-based care, which was associated with acute stress, depression, and anxiety. | Extra bonus policy, reassurance from the management, understanding and communication from the leaders were useful. 91% of the survey respondents found it useful. | Those who did not find the services offered by the hospital might have mental health issues. |
| 23 | Blake H, Yildirim M, Wood B, Knowles S, Mancini H, Coyne E, Cooper J. COVID-Well: Evaluation of the Implementation of Supported Wellbeing Centres for Hospital Employees during the COVID-19 Pandemic. Int J Environ Res Public Health. 2020 Dec 15;17(24):9401. | Supported Wellbeing Centres (relaxing spaces with comfortable seating, relaxing music, low-level lighting, plants and an aromatherapy pod. Refreshments were available, and for a limited time period in the early days of opening there were charitable donations for employees (including personal care packages, wash bags, toiletries, snacks, and washable uniform). - wellbeing buddies ( 134 buddies were trained in PFA). Also, well-being buddies ( 134 buddies were trained in PFA) provided psychological and emotional support at the centers through active listening. | Improved environment/basic needs (Therapy & rehabilitation) | Acute hospital trust: Site A and Site B | Positive<br>Many positive comments on the center`s impacts on staff wellbeing. | Offered quiet rest and recuperation, suggesting an ongoing need for time-out facilities and rest spaces for hospital workers. Other common reasons for access were for social contact and peer support, or to access wellbeing resources and signposting from a wellbeing buddy. Wellbeing scores of those accessed the centres were highger than those who did not. | Significant human resource and financial cost in running the centres for a longer period of time. Alternative approaches are needed in the long-run. |

| | | | | | | | |
|---|---|---|---|---|---|---|---|
| 24 | Ripp J, Peccoralo L, Charney D. Attending to the Emotional Well-Being of the Health Care Workforce in a New York City Health System During the COVID-19 Pandemic. Acad Med. 2020 Aug;95(8):1136-1139. | Personnel: Offered childcare, free parking and bike rental as well as hotel and accomodation for staff on top of the consultation meetings.<br><br>Organizational: "All hands on deck" approach. Also taskforce was created work group strategy during the pandemic to review and operationalize plans for priority areas. Task force maintained close communication with related stakeholders (system, hospital, department heads and hospital workers). Task force includes reps from office of wellbeing and resilience, HR, employee assistance program, department of psychiatry, nursing, social work and others. "Wellness messages" as suggested by the trained mental health experts in addition to daily to weekly conference calls. Simple self-care sources ranging to virtual support group were provided. | Improved environment/basic needs (Therapy & rehabilitation) | Mount Sinai Health System: largest hospital system in New York City | N.A | Offered various services to allow each and every member of the hospital to benefit to certain degree. Added to the previously exisitng psychological help (employee assistance program, psychiatry services, spiritual care), which includes simple self-care resources (e.g mindfulness activities) to virtual support groups facilitated by social worker | N/A |
| 25 | Blake H, Bermingham F, Johnson G, Tabner A. Mitigating the Psychological Impact of COVID-19 on Healthcare Workers: A Digital Learning Package. Int J Environ Res Public Health. 2020;17(9):2997. | Provision of educational package: Psychosocial e-learning package including lessons around 7 themes; psychological impacts, supportive teams, communication; social support; self-care; managing emotions was developed and provided. | Educational package (Training and Information support) | Healthcare workers at hospital and healthcare students | Positive<br>Immediate adoption within their health and wellbeing provisions of health care providers. High user satisfaction with content, usability and utility. | The learning package was offered free of charge and was accessible to everybody (including low-resource countries' HCWs).<br><br>Managers and employees of any hospital and organization can access and use it tailoring to their own needs.<br><br>Interactive educational tool, format is simple. | Periodical app update is required.<br><br>The cost is beared by the management<br><br>Not individually tailored thus those seeking more individual services might not find this helpful. |
| 26 | Robles, R., Palacios, M., Rangel, N., Real, T., Becerra, B., Fresán, A., Vega, H., Rodríguez, E., Durand, S., & Madrigal, E. (2020). A qualitative assessment of psycho-educational vid-eos for frontline COVID-19 healthcare workers in Mexico. Salud Mental, 43(6), 311-318 | Provision of educational package: The initiative includes two sets of concise psycho-educational videos tailored to address the educational requirements of HCWs. These videos focus on widely recognized evidence-based techniques aimed at promoting self-care, providing psychoeducation on burnout, and advocating preventive measures based on cultivating healthy habits. | Educational package (Training and Information support) | One center in Guadalajara, Jalisco; two are in the *Estado de México*, and two in Mexico City | Positive<br>Video contents were very beneficial, relevant and applicable to the workplace and even in their everyday personal and family life | Accessible for all HCWs (including administrative workers)<br>Remotely accessible. | Some videos might be too long for HCWs to watch in one sitting |

| | | | | | | | |
|---|---|---|---|---|---|---|---|
| 27 | Maldonato NM, Bottone M, Chiodi A, Continisio GI, De Falco R, Duval M, Muzii B, Siani G, Valerio P, Vitelli R, et al. A Mental Health First Aid Service in an Italian University Public Hospital during the Coronavirus Disease 2019 Outbreak. Sustainability. 2020; 12(10):4244. | Hotline service for the HCWs was established through a systematic approach with expanded teams.<br>1. Management team: handled the bereaucratic issues and liasion to hte hospital administration<br>2. Clinical area team: provided the psychological apsychiatric support<br>3. Communication are team: disseminated information about the service to the public<br>4. Research area: Tasked with an assessment procedure | Mental health consultation | Italian university public hospital | N.A<br>Due to the emergency nature of the service, it was not possible to implement a complex system that allow us the real effectiveness of our actions. | Service was available at anytime, can leave voice messages to the hotline consultants. | The hotline consultants' team solely offered psychological first aid without further background information, lacking connections to local psychologists or the provision of additional resources like applications (apps) for employee support. |
| 28 | Chen Q, Liang M, Li Y, et al. Mental health care for medical staff in China during the COVID-19 outbreak [published correction appears in Lancet Psychiatry. 2020 May;7(5):e27]. Lancet Psychiatry. 2020;7(4):e15-e16. | Detailed psychological intervention plan was developed. Three services were provided: 1.online courses for HCWs to deal with common psychological problems; 2.hotline service; 3. groups activities | Mental health consultation | Second Xiangya Hospital | Positive<br>Implementation of the psychological intervention services encountered obstacles, thus interventions were adjusted to followings: 1. Provision of rest area and food for the staff and video recording of daily activities for family members; 2. pre-job training and hospital security staff assistance for dealing with psychological probems in patients; 3. development of a detailed rule on use and management of protective equipment; 4. Regular visits of psychological counsellor to listen to stories of staff at work. The adjusted intervention made medical staffs feeling at home. | The hospital adjusted the services after hearing out the HCW's opinion through survey. They added place to rest, food and daily living supplies, video recording to send to their families as requested. | Medical staff were not willing to participate in group psychological activities. Besides staff were obviously exhausted and irritated but declined the psychological help (might have needed more awareness activities). |
| 29 | Viswanathan R, Myers MF, Fanous AH. Support Groups and Individual Mental Health Care via Video Conferencing for Frontline Clinicians During the COVID-19 Pandemic. Psychosomatics. 2020;61(5):538-543. | Providing assistance through peer support groups, individual video conference sessions for counselling, and support group conference calls via telephone for HCWs seeking support and guidance. | Mental health consultation | University Hospital of Brooklyn | Positive<br>Group interventions help reach a larger number of people with a limited number of mental health professionals and can offer some additional healing elements that individual approaches do not. | Can reach a larger number of people with limited mental health professionals. Having professional facilitators helped to run the group sessions smoothly. | Cannot fix problems of people with serious mental health issues. |

| | | | | | | | |
|---|---|---|---|---|---|---|---|
| 30 | Mira JJ, Vicente MA, Lopez-Pineda A, et al. Preventing and Addressing the Stress Reactions of Health Care Workers Caring for Patients With COVID-19: Development of a Digital Platform (Be + Against COVID). JMIR Mhealth Uhealth. 2020;8(10):e21692. | An application designed to offer individualized resources for HCWs, catering to their specific needs and providing access to a range of supportive materials and tools. | Mental health consultation | HCW from different countries, mostly from Spain | Positive<br>"Self-Report Acute Stress Scale" was the most visited resource since the application launch. | Available in 3 languages (Spanish, English, Brazilian Portuguese); Available for anyone with internet ; Self-report scale is available.<br><br>Professionals from multisector were involved in the development of the app; Both mobile app and website (mobile app is easier to use) are available. | Not tailored to specific group or population, some content might be too broad. |
| 31 | Kang L, Li Y, Hu S, et al. The mental health of medical workers in Wuhan, China dealing with the 2019 novel coronavirus. Lancet Psychiatry. 2020;7(3):e14. | A psychological intervention team was established, structured into four primary levels of officials:<br>1. Response Team: Responsible for immediate interventions and initial responses to psychological concerns.<br>2. Technical Support Team: Provides specialized support and expertise in handling psychological interventions.<br>3. Medical Team: Comprising medical professionals who offer specific medical and psychological assistance.<br>4. Hotline Team: Manages and operates the hotline service, offering direct support and guidance to individuals seeking assistance. | Mental health consultation<br><br>Group activities/peer support | HCWs in Wuhan province, China | Positive<br>Hundreds of medical workers who received this intervention (online platforms with medical advice have been provided to share information on how to decrease the risk of transmission between the patient in medical settings, which aims to eventually reduce the pressure on medical workers) were satisfied. This intervention provision is expanding to more people and hospitals. | Allows to have complex response team and increases the work efficiency which itself will guarantee a better-quality service to HCWs. | |
| 32 | Monette DL, Macias-Konstantopoulos WL, Brown DFM, Raja AS, Takayesu JK. A Video-based Debriefing Program to Support Emergency Medicine Clinician Well-being During the COVID-19 Pandemic. West J Emerg Med. 2020;21(6):88-92. | A video-based emergency department debriefing program supported to enhance the well-being and support of emergency clinicians. | Mental health consultation | Massachusetts General Hospital | Positive<br>The debriefing provided a platform to discuss unmet needs to improve self-care and build resilience. | The video-based debriefing was helpful in these 4 aspects:<br>1. facilitators created a safe environment,<br>2. debriefing with members of the same role group;<br>3. facilitators were trusted colleagues;<br>4. the Zoom platform was easy to use | The program might be efficient only for those who are comfortable with sharing their emotional reactions with peers and showing vulnerabilities. |

| | | | | | | | |
|---|---|---|---|---|---|---|---|
| 33 | Feinstein RE, Kotara S, Jones B, Shanor D, Nemeroff CB. A health care workers mental health crisis line in the age of COVID-19. Depress Anxiety. 2020;37(8):822-826. | The HCW Mental Health COVID-19 Hotline was developed to provide crisis counselling by utilizing the expertise of psychiatrists, clinical social workers, psychiatric residents, and volunteer mental health professionals. | Mental health consultation | Dell Medical School Community and Seton Hospitals and Affiliates | Positive<br>13-steps inclusive model for hotline led to success was shared. | Hotline service was developed using the already existing resources. The team included multidisciplinary members (clinical social workers, faculty and staff from Psychiatry department, medical school leadership and administrator of the service).<br><br>Training and educational materials were provided to the service providers. Follow-up surveys were administered 2 weeks after the service was launched. | |
| 34 | Geoffroy PA, Le Goanvic V, Sabbagh O, et al. Psychological Support System for Hospital Workers During the Covid-19 Outbreak: Rapid Design and Implementation of the Covid-Psy Hotline. Front Psychiatry. 2020;11:511. | The "COVID-PSY Hotline" refers to a psychosocial intervention hotline established specifically to provide support and assistance to individuals facing psychological challenges or distress related to the COVID-19 pandemic. | Mental health consultation | All hospital workers in Paris | Positive<br>The hotline seems to benefits all hospital professionals that all appeared psychologically affected. | The hotline service was available to all staff, including both frontline and non-frontline workers, and was swiftly implemented within three days of the onset of COVID-19.<br><br>It involved multiple psychologists and volunteers ready to assist, providing staged help by initially identifying symptoms and then referring individuals to psychologists if necessary. This intervention complemented existing helplines and local psychological services available within health institutions. | Caller feedback and satisfaction were not documented, rendering it challenging to assess the effectiveness of the intervention.<br><br>The opportunity cost incurred by staff and resources involved in this initiative could not be accurately measured. |
